# CXCL5 neutralization mitigates cancer cachexia by disrupting CAF-cancer cell crosstalk

**DOI:** 10.1186/s12929-025-01192-0

**Published:** 2025-12-15

**Authors:** Hyun-Jun Kim, Seon-Wook Kim, Jun-Hyeong Kim, Sang-Hoon Lee, Kyoung-Hwan Joo, Soo Young Lee, Hyunju Lee, Jung-Joon Min, Sang-Hee Cho, Da-Woon Jung, Darren R. Williams

**Affiliations:** 1 New Drug Targets Laboratory, Department of Life Sciences, College of Life Sciences and Medical Engineering, Gwangju Institute of Science and Technology, Gwangju, Republic of Korea; 2 Department of Surgery, Chonnam National University Hwasun Hospital, Chonnam National University Medical School, Hwasun, Republic of Korea; 3 School of Electrical Engineering and Computer Science, Gwangju Institute of Science and Technology, Gwangju, Republic of Korea; 4 Division of Hematology-Oncology, Department of Internal Medicine, Chonnam National University Hwasun Hospital, Chonnam National University Medical School, Hwasun, Korea

**Keywords:** Cancer cachexia, Cancer-associated fibroblasts, CXCL5, Crosstalk, Skeletal muscle wasting, Tumor microenvironment

## Abstract

**Background:**

Advanced metastasis produces cachexia, a complex skeletal muscle wasting syndrome that accounts for one-third of patient deaths. There is currently no approved drug therapy for cancer cachexia. Cancer-associated fibroblasts (CAF) within tumors have been hypothesized to contribute to cachexia, but the detailed mechanism(s) are unknown.

**Methods:**

Myotubes were treated with conditioned media (CM) from CAF or CAF activated by cancer cells. Upregulated chemokines in the cancer-activated CAF CM were identified by cytokine array. The effects of chemokine neutralization were investigated using in vitro myotube cultures and in vivo mouse models. The mechanism of action was characterized by in vivo RNA Seq and validated in human muscle cells. Immunostaining delineated the chemokine expression pattern in a patient tumor type highly associated with cachexia.

**Results:**

Cancer-activated CAF induced myotube atrophy. CXCL5 was as the major chemokine highly upregulated in the cancer-activated CAF*.* CXCL5 treatment alone induced myotube atrophy and inhibited myogenic ERK1/2 signaling, similar to cancer-activated CAF treatment. CXCL5 neutralization inhibited cachexia in mice co-injected with HCT 116 colon cancer cells and CAF. RNA Seq showed that CXCL5 neutralization upregulated hypertrophy-related PI3K-AKT-MyoG signaling and remodeled the muscle ECM. CXCL5 neutralization ameliorated muscle wasting induced by CXCL5 and IL-6 co-treatment, and also prevented atrophy in cancer-activated CAF CM-treated human myotubes. CAF were the major detectable source of CXCL5 in a patient tumor highly associated with cachexia.

**Conclusion:**

CAF contribute to cachexia via cancer cell crosstalk that upregulates CXCL5 secretion. CXCL5 neutralization offers a novel therapeutic approach to maintain muscle mass in cancer patients.

**Supplementary Information:**

The online version contains supplementary material available at 10.1186/s12929-025-01192-0.

## Introduction

Cancer cachexia is defined as weight loss in patients exceeding 5% within 6 months [1]. Cachexia inhibits the effectiveness of chemotherapy, immunotherapy, and the ability to tolerate major surgery [2, 3]. Up to 80% of patients develop cachexia depending on cancer type [4]. Cardiac and respiratory system failure can occur when weight loss reaches 25–30%. Cachexia is thought to account for more than 30% of all cancer deaths [5].

Cancer cachexia is a complex paraneoplastic syndrome that does not respond to conventional nutritional support. There are three distinct phases: precachexia, cachexia and refractory cachexia [5, 6]. Research has focused on characterizing the major causative mechanisms and developing effective therapeutics. However, there is currently no defined standard of care available to counter tissue wasting [5]. Treatment with appetite stimulants can reverse the loss of fat, but recovering wasted skeletal muscle is more challenging [7]. This difference can be significant, because skeletal muscle parameters, such as muscle fiber atrophy, myosteatosis, fibrosis, grip strength, and inflammation have been identified as independent prognostic factors for reduced cancer-specific, disease-free, or overall survival in patients [8, 9].

Although the major catabolic drivers of muscle wasting in cachectic patients are not fully characterized [10], a number of tumor-derived inflammatory cytokines have been shown to promote cancer cachexia, such as tumor necrosis factor α (TNFα) and interferon-γ (IFN- γ) [11]. This supports the definition of cancer cachexia as a chronic inflammatory disease [12]. Studies with cancer cells have identified other secreted factors termed ‘cachexokines’ that contribute to cachexia. These include bridging integrator 1, multiple inositol-polyphosphate 1, glucosidase alpha acid, and ataxin-10 [11]. Cachexokines have been showed to induce muscle atrophy and disrupt normal lipid metabolism [11]. Other serum-born factors have been shown to contribute to cancer cachexia, including modified ceramides, exosomes and microvesicles [13, 14].

Tumors can be viewed as abnormal organs formed by a heterogeneous cell population composed of cancer cells and multiple non-cancer cell types, such as immune cells, neutrophils, cancer-associated fibroblasts (CAF), adipocytes, and blood or lymph endothelial cells. Together, these cells makeup the tumor microenvironment [15]. The majority of research to characterize the molecular mechanisms driving cancer cachexia have focused on the tumor as a whole and utilized cancer cell lines for in vitro studies. There have been relatively few studies of the contribution of non-cancer tumor microenvironment cells to cancer cachexia.

CAF are an abundant cell type within the tumors. They form the tumor stroma tissue and play crucial roles in tumorigenesis and metastasis via a complex network of intercellular communication with cancer cells termed ‘crosstalk’ [16, 17]. However, the potential role of CAF-cancer cell crosstalk in skeletal muscle wasting has not been investigated. Identifying the major cachexokines derived from CAF-cancer cell crosstalk and their mechanism of action can increase our understanding of this complex wasting disorder and provide an opportunity to develop novel therapeutic strategies. In this study, the influence of CAF on muscle wasting was studied in vitro using cancer-activated CAF CM-treated myotubes, cytokine arrays, humanized animal models of cancer cachexia, in vivo RNA-Seq, human donor muscle cells, and patient-derived tumor samples.

## Materials and methods

### Cell culture

The C2C12 murine skeletal muscle myoblast cell line was purchased from Koram Biotech. Corp (Republic of Korea). Myoblasts were cultured in growth media consisting of (Dulbecco’s Modified Eagle’s Medium (DMEM), 10% fetal bovine serum (FBS) and 1% penicillin and streptomycin (PenStrep). The myoblasts were differentiated into myotubes when reaching approximately 90% confluence by culturing in differentiation media (DMEM, 2% horse serum (HS) and PenStrep) for 72 h*.* Myotubes were defined as elongated cells containing more than 3 nuclei.

Patient-derived colon cancer CAF were purchased from Neuromics (MN, USA) and cultured in F-media (DMEM:F-12 = 3:1), 10% FBS and 1% PenStrep. HCT 116 human colon carcinoma cells and CCD-18Co normal human colon fibroblasts (NF) were purchased from the American Type Culture Collection (VA, USA). HCT 116 cells were cultured in DMEM supplemented with 10% FBS and 1% PenStrep. CCD-18Co cells were cultured in F-media, 10% FBS and 1% PenStrep.

Conditioned media (CM) were harvested from HCT 116, CCD-18Co and CAF at approximately 100% confluence with serum free media for 24 h. CAF CCM were harvested with 50% of HCT 116 CM and 50% of F-media for 24 h. When preparing the CM from normal fibroblasts, CAF, and cancer cells, the CM was diluted with serum free media to a 1:1 ratio, and HS was the added to make a 2% final concentration, which is the same as the differentiation media for the myotubes. CM was treated to the differentiated myotubes for 72 h. CXCL5 neutralizing antibody MAB-254 was purchased from R&D system (MN, USA) and CXCR2 antagonist SSB225005 was obtained from MedChemExpress (NJ, USA).

Human donor skeletal myoblasts were purchased from Thermo-Fisher Scientific (MA, USA). The myoblasts were thawed in a water bath, centrifuged at 180 g for 5 min at room temperature, and washed with 20 mL of differentiation media. Myoblasts were then re-suspended in differentiation media, followed by seeding onto 12 well plates (density = 4.8 × 10^4^ cells/well) and used for experiments 48 h later.

Myotube diameter was measured with ImageJ 1.52 software (National institutes of Health, MD, USA) using DIC captured images (Olympus CKX41). At the end point of the experiment, 5 pictures were taken of each well and the diameter of 30 myotubes was measured in each image.

### Isolation of CAF from patient colon cancer tissue

CAF were isolated from tumor tissue obtained from patients undergoing tumor resection surgery for colon cancer (supplied by Chonnam medical school, Gwangju, Republic of Korea (IRB approval number: CNUHH-2021-217)). Donated tumor tissues were dissected under sterilized conditions and CAF were harvested using digestion buffer (collagenase A (Roche, USA) and dispase II (Wako, Japan)). CAF were tested for mycoplasma three times before co-culture and transplantation studies using the Mycoplasma Removal Reagent kit (Capricorn, Germany). The fibroblastic origin of the CAF was confirmed using vimentin staining (Supplementary Fig. 1A).

### Immunocytochemistry

C2C12 murine myotubes and human donor skeletal myotubes were visualized by myosin heavy chain immunocytochemistry to measure myotube diameter and the differentiation and fusion indexes [18]. In brief, myotubes were fixed with 3.7% formaldehyde solution, permeabilized using 0.2% Triton X-100, and blocked with 1% BSA in PBST. Primary antibody was incubated overnight at 4 °C and the secondary antibody was incubated for 1 h at room temperature. Myotubes were counterstained with 1 µg/mL of DAPI in PBS.

### Western blotting

To harvest protein lysates, cells were rinsed with PBS to remove the culture media and ice-cold lysis buffer containing protease inhibitor cocktail (Sigma-Aldrich, MA) was added to the cells cultured in 10 cm dishes. Cell lysates were harvested with a cell scraper and transferred to a microfuge tube. The tubes were incubated on ice for 15 min and centrifuged at 13,000 rpm for 15 min. The supernatants were then transferred to a new 1.5 mL tube.

Dissected tissues were chopped into small pieces using dissection scissors, transferred to a microfuge tube and incubated with ice-cold lysis buffer. The incubation and centrifugation steps were the same as for the cell lysates.

Protein lysates harvested from cells or tissues were quantified using the Bradford reagent (Bio-Rad, USA, CA). Proteins were separated by electrophoresis with 10–12% SDS-PAGE, transferred onto PVDF membranes (Merck, Germany), and blocked with 5% bovine serum albumin in TBST. Incubation with the primary antibody was carried out overnight at 4 °C. Secondary antibody incubation was 30 min at RT. Densitometry of the protein bands was carried out using ImageJ 1.52 software. Primary and secondary antibodies used in this study are listed in Tables 1 and 2.

**Table 1:** Primary antibodies used in this study

Primary antibody	Clone	Company	Cat. No	Dilution
GAPDH	Monoclonal	Santacruz	Sc-365062	1:1000
MYH2	Monoclonal	Santacruz	Sc-53095	1:1000
Atrogin-1	Monoclonal	Abcam	Ab168372	1:1000
MuRF-1	Monoclonal	Santacruz	Sc-398608	1:500
Phoshpo-p44/42 MAPK (ERK1/2) (Thr202/Tyr204)	Monoclonal	CST	9101S	1:1000
p44/42 MAPK (ERK1/2)	Monoclonal	CST	9102S	1:1000
Phospho-PI3 Kinase p85 (Tyr458)/p55 (Tyr199) (E3U1H)	Monoclonal	CST	17366S	1:1000
PI3 Kinase p85α (6G10)	Monoclonal	CST	13666S	1:1000
Phospho-AKT ( Ser473)	Monoclonal	CST	9271S	1:1000
AKT	Monoclonal	CST	9272S	1:1000
P-IκBα (Ser32/36)	Monoclonal	CST	9246S	1:1000
IκBα (44D4)	Monoclonal	CST	4812S	1:1000
NF-κB p65 (D14E12)	Monoclonal	CST	8242S	1:1000
Puromycin (12D10)	Monoclonal	Merck	MABE-343	1:2000
Alpha-tubulin	Polyclonal	Invitrogen	PA5-29444	1:1000
Vimentin	Monoclonal	CST	5741S	1:100
Cytokeratin	Polyclonal	Abcam	Ab9377	1:100
CXCR2	Polyclonal	Invitrogen	PA5-100951	1:1000
Laminin	Polyclonal	Abcam	Ab11575	1:1000
Mouse IgG1 Isotype control	Monoclonal	R&D systems	MAB002	120 μg/kg
Human CXCL5/ENA-78 Antibody	Monoclonal	R&D systems	MAB254-500	120 μg/kg

**Table 2:** Secondary antibodies used in this study

Secondary antibody	Conjugated use	Company	Cat. No	Dilution
Horse anti-Mouse IgG HRP	HRP	CST	#7076	1:10,000
Goat anti-Rabbit IgG HRP	HRP	CST	#7074	1:10,000
Alexa FluorTM 488 Goat anti-mouse IgG(H + L)	Alexa Fluor 488	Invitrogen	A11001	1:200
Goat anti-rabbit IgG H + L Chain Antibody DyLight® 488 Conjugated	Dylight 488	Bethyl	A120-101D2	1:1000
Goat anti-rabbit IgG H + L Chain Antibody DyLight® 594 Conjugated	Dylight 594	Bethyl	A120-101D4-4	1:1000
Goat anti-mouse IgG H + L Chain Antibody DyLight® 594 Conjugated	Dylight 594	Bethyl	A90-116D4	1:200

### Real-time qPCR

mRNA expression levels of the genes of interest were measured with the StepOnePlus Real-Time PCR System (Applied Biosystems, UK), as previously described [19]. In brief, the AccuPower RT PreMix (Bioneer, Republic of Korea) was used to synthesize cDNA from total RNA*.* Real-time PCR (qPCR) was carried out following the manufacturer’s instructions, with modifications as follows: PCR was performed in triplicate with the 20 μL 2X Power SYBR Green PCR Master Mix (Enzynomics, Republic of Korea) containing 200 nM of the targeted primers and 1 μL of cDNA. The primers used in this study are described in Table 3. A specific cDNA sample was included in each run and used as a reference between the runs. GAPDH expression was used for normalization when measuring the expression levels of the genes of interest.

**Table 3:** Primers used in this study

Primer name	Primer sequence		Size (bp)	Accession number
Atrogin-1	Forward	CAGAGAGCTGCTCCGTCTCA	178	NM_026346
	Reverse	ACGTATCCCCCGCAGTTTC		
MuRF-1	Forward	CCGAGTGCAGACGATCATCTC	198	NM_001039048
	Reverse	TGGAGGATCAGAGCCTCGAT		
MyoG	Forward	AGCGCAGGCTCAAGAAAGTG	181	NM_031189
	Reverse	CCGCCTCTGTAGCGGAGAT		
GAPDH	Forward	CTCCACTCACGGCAAATTCA	120	NM_001289726
	Reverse	GCCTCACCCCATTTGATGTT		
Dkk2	Forward	CGCAACCATGGTCACTATTCC	150	NM_020265
	Reverse	GAAGTGGCGAGCACAACAAA		
Col6a5	Forward	TGTAGTGGTCGGGTTCGACAT	250	NM_001167923
	Reverse	ACGGACTGTGACGTGCAACA		
Pf4	Forward	GTTTCTGCCAGCGGTGGTT	100	NM_019932
	Reverse	ATGGATCCCAGAGGAGATGGT		
Dusp2	Forward	CGAGGCGGTTTCAAAAGCT	110	NM_010090
	Reverse	ACCCTCGGGTCAGAGTTGCT		
Gatm	Forward	AGCTACAGCTTCCTCCCGAAA	150	NM_025961
	Reverse	AATGGTGGCACACAGGCATT		
Sphk1	Forward	GCCAGTGCCTTCTCATTGGA	150	NM_001172472
	Reverse	CCCGCACGTACGTAGAACAGA		
Serpina3n	Forward	GGCTCAACCAGCCAAAGGA	150	NM_009252
	Reverse	GACGAGGCTGCTGGAAGTCT		
Cxcl1 (human)	Forward	CACTCAAGAATGGGCGGAAA	104	NM_001511.4
	Reverse	CCCTTCTGGTCAGTTGGATTTG		
Cxcl2 (human)	Forward	CTCAAGAATGGGCAGAAAGCTT	101	NM_002089.4
	Reverse	CCTTCTGGTCAGTTGGATTTGC		
Cxcl3 (human)	Forward	CCGAAGTCATAGCCACACTCAA	101	NM_002090.3
	Reverse	GCTCCCCTTGTTCAGTATCTTTTC		
Cxcl5 (human)	Forward	TTCATCCCAAAATGATCAGTAATCTG	100	NM_002994.5
	Reverse	CAAATTTCCTTCCCGTTCTTCA		
Cxcl6 (human)	Forward	TTGCACTTGTTTACGCGTTACG	103	NM_002993.4
	Reverse	GGCTACCACTTCCACCTTGGA		
Cxcl7 (human)	Forward	ATGCTGAACTCCGCTGCAT	100	NM_002704.3
	Reverse	TGGTTGCAATGGGTTCCTTT		
Cxcl8 (human)	Forward	TCAGAGACAGCAGAGCACACAA	100	NM_000584.4
	Reverse	GGCCAGCTTGGAAGTCATGT		
IL-6 (human)	Forward	AGTGGCTGCAGGACATGACA	104	NM_000600.5
	Reverse	TCTGAGGTGCCCATGCTACA		
GAPDH (human)	Forward	CTGCACCACCAACTGCTTAGC	107	NM_002046.7
	Reverse	TCTTCTGGGTGGCAGTGATG		

### Enzyme-linked immunosorbent assay (ELISA)

Duoset® ELISA Development system kits for human CXCL5/ENA-78 (Catalog #: DY008) and human IL-6 (Catalog #: DY206) were purchased from R&D Systems (MN, USA). ELISA was carried out in accordance with the manufacturer’s instructions.

### Cytokine antibody array

Cytokine levels were measured in HCT 116 CM, CAF CM and CAF CCM using the human cytokine antibody (Ab) array C3 (Raybiotech, GA, USA), following the manufacturer's instructions. Quantification of cytokine levels was carried out using ImageJ 1.52 software.

### Measurement of protein synthesis

The surface sensing of translation (SUnSET) assay was used to assess protein synthesis, as described previously [20]. In summary, 1 μg/mL puromycin was treated to the myotube cultures and cell lysates were harvested 10 min later. Lysates were then processed for western blotting using the anti-puromycin 12D10 antibody (MABE343, Sigma-Aldrich, MA).

### Animal studies

Animal studies were carried out under the guidance of the Institute for Laboratory Animal Research Guide for the Care and Use of Laboratory Animals, and approved by the Gwangju Institute of Science and Technology Animal Care and Use Committee (study approval number GIST-2021-098). Animal studies have been approved by the appropriate ethics committee and have therefore been performed in accordance with the ethical standards laid down in the 1964 Declaration of Helsinki and its later amendments. Animals were purchased from Orient Bio (Republic of Korea).

### Humanized model of colon cancer-induced cachexia

Ten week old female NOD-SCID mice were xenografted with 1 × 10^6^ HCT 116-Luc2 alone *(*ATCC, VA, USA) or 1 × 10^6^ of HCT 116 and 2 × 10^6^ human CAF cells subcutaneously into the right flank (n = 7 per group). To inhibit CXCL5, 120 μg/kg of neutralizing antibody (R&D Systems, MAB-254, clone 33160, MN, USA) and IgG1 monoclonal antibody (R&D Systems, MAB-002, clone 11711, MN, USA) were treated for 3 d prior to transplantation via intraperitoneal (IP) injection. Evidence of neutralization was provided by the company supplier and the study by Chen, et al., which demonstrated that this antibody was neutralizing in a model of hind limb ischemia [21]. Tumor bearing mice were measured for tumor growth using the IVIS system (Caliper IVIS Kinetic In Vivo Optical Imaging System, MA, USA). Luciferin (Promega, WI, USA) was dissolved in DPBS and injected at a concentration of 6 mg/mouse.

### Hanging tolerance test

Mice were tested for their hanging tolerance prior to sacrifice, using a height of 50 cm with a cushioned drop. The hanging tolerance was recorded one time for each mouse.

### Muscle dissection and histology

Mice were anesthetized with 22 mg/kg ketamine (Yuhan, Republic of Korea) and 10 mg/kg xylazine (Bayer, Republic of Korea) by IP prior to sacrifice. The quadriceps, gastrocnemius, tibialis anterior (TA) or soleus muscles were dissected and weighed. Muscles were fixed by overnight incubation with 3.7% paraformaldehyde at 4 °C, embedded into paraffin solution and stored at 4 °C. Paraffin sectioning and hematoxylin and Eosin (H&E) staining was carried out by the Animal Research Facility of the Gwangju Institute of Science and Technology, Republic of Korea, using an H&E kit (Merck, Germany). Muscle fiber cross sectional area was measured with the ImageJ 1.52 software (National institutes of Health, MD, USA). 100 myofibers were measured in 5 captured images for each muscle. Mice were anesthetized with 2.5% isoflurane (Hana, Republic of Korea) for 3 min before sacrifice. The quadriceps, gastrocnemius, TA (TA), and soleus muscles were dissected and weighed. For immunohistochemistry, dissected muscles were sequentially embedded in 10%, 20% and 30% sucrose solution every 24 h at 4 ºC, and then embedded into Cryo-OCT block for 24 h at 4 ºC, followed by storage at − 80 °C. Muscle sections were obtained using a CM 1860 cryostat (Leica) and mounted with DAPI Mount (Invitrogen).

### Immunohistochemistry

Skeletal muscle sections was carried out using anti-laminin antibody (Abcam, Cambirdge, UK) and anti-CXCR2 (Invitrogen, Massachusetts, U.S). Counterstaining was conducted with a 1 μg/mL of DAPI solution. TA muscles were sectioned at 10 μM thickness to measure the cross-sectional area (CSA) and the proportion of CXCR2 positive fibers. Sections were visualized with fluorescence microscopy (Leica DM 2500). Muscle fiber CSA and myofiber distribution were measured with the ImageJ 1.48 software (NIH, USA).

### In vivo RNA-Seq

RNA samples were harvested from the quadriceps muscles of treated mice. RNA-Seq was carried out by Macrogen Inc., Republic of Korea. Sample QC was performed with FastQC v0.11.7 (http://www.bioinformatics.babraham.ac.uk/projects/fastqc/). For illumina paired-end or single-end trimming process, Trimmomatic 0.38 software (http://www.usadellab.org/cms/?page=trimmomatic) was used for the various parameters. HISAT2 version 2.1.0, Bowtie2 2.3.4.1 (https://ccb.jhu.edu/software/hisat2/index.shtml) provided sequencing read mapping for the Hierarchical Graph FM index (HGFM). The potential transcript assembly tool was StringTie version 2.1.3b (https://ccb.jhu.edu/software/stringtie/).

### Cytokine treatment model of cachexia

Recombinant CXCL5 at a dose of 40 ng/kg and IL-6 at a dose of 80 ng/kg were treated to 10-week-old female C57BL/6 mice three times a week for four weeks via IP delivery. 72 h prior to cytokine delivery, mice were pre-treated with the IgG1 and CXCL5 neutralization antibody at a dose of 120 µg/kg. Throughout the four-week period, neutralization antibody was co-administered with the cytokines three times per week.

### Preparation and immunohistochemical analysis of patient colon tumor tissue

Colon tumor tissues were provided by Chonnam Medical School, Gwangju, Republic of Korea (IRB approval number: CNUHH-2021-217). The donor’s age, gender and stage are provided in Table 4. The tissues were fixed in 3.7% paraformaldehyde for 24 h at 4 °C for paraffin sectioning. Slide sections were deparaffinized by xylene and serial diluted ethanol, permeabilized with 0.1% Triton-X, blocking and antibody treatment was performed with 3% BSA in 0.05% Triton-X. Prolong gold antifade reagent with DAPI (Thermofisher, USA) was used for mounting. Immunofluorescence images were obtained with a DMI 3000 B microscope, and analyzed using the ImageJ 1.52 software.

**Table 4:** Age, gender and stage of tumor tissue donors

Number	Sex	Age	Stage
1	Female	86	3
2	Male	71	1
3	Male	71	2

### Statistical analysis

Statistical significance was determined using the Student’s *t*-test. A *p* value of less than 0.05 was considered as significant. Unless otherwise mentioned, all data shown are representative of more than 3 experimental repeats and the graph error bars are standard deviation or standard error, as indicated.

## Results

### Cancer-stimulated CAF induce myotube atrophy and upregulate CXCL5

**Fig. 1:**
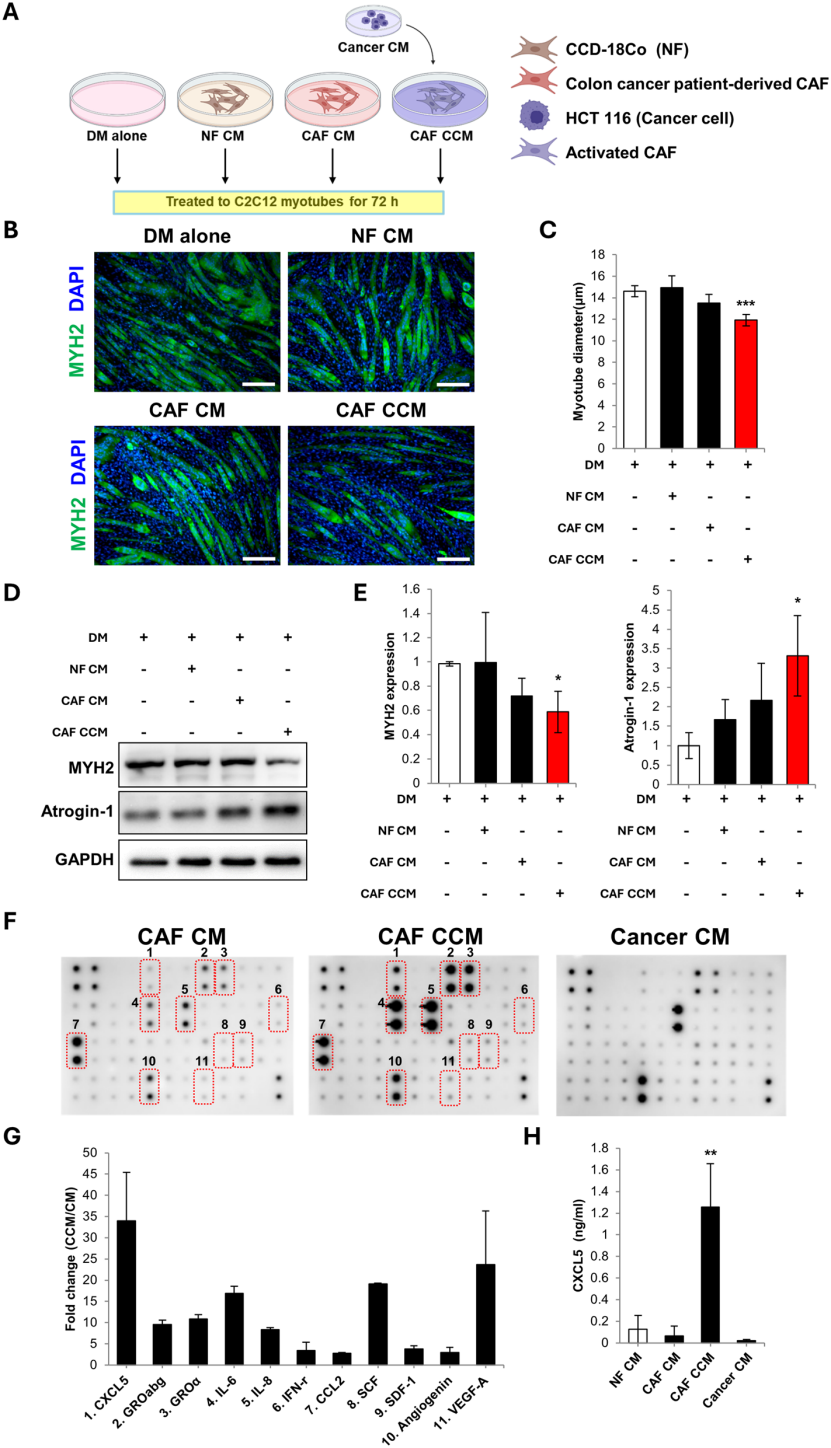
**A** Schematic of the protocol to compare the effects of conditioned media (CM) from CCD-18Co human normal colon tissue fibroblasts (NF CM), primary human colon cancer-associated fibroblasts (CAF CM), and human HCT 116 colon cancer cell-stimulated CAF CM (CAF CCM) on C2C12 myotube wasting. **B** Myosin heavy chain 2 (MYH2) immunostaining of C2C12 myotubes cultured in normal myotube differentiation media (DM) and treated with NF CM, CAF CM, and CAF CCM for 72 h (scale bar = 150 μm). **C** Calculation of mean myotube diameter. **D** Western blot analysis of MYH2 and atrogin-1 expression in the treated myotubes. **E** Densitometry of MYH2 and atrogin-1 expression relative to GAPDH. **F** Cytokine array analysis CAF CM, CAF CCM, and HCT 116 cancer cell CM. Red boxes and numbers indicate upregulated cytokines in the CAF CCM compared to CAF CM. **G** Quantification of the fold-change for the upregulated cytokines. **H** ELISA-based detection of CXCL5 in NF CM, CAF CM, CAF CCM, and HCT 116 cancer cell CM. The CAF CM and CCM values are the mean obtained from three sources of CAF: two derived from patients and one provided commercially. All experiments were conducted 3 times independently and the values are indicated as the mean ± SD. For **C** and **E**: * = *p* < 0.05 and *** = *p* < 0.001 compared to DM. For **H**: ** = *p* < 0.01 compared to CAF CM

The potential role of colon carcinoma CAF in skeletal muscle atrophy were initially investigated in vitro using myotube cultures. Colon cancer was selected because it is a tumor type highly associated with cachexia [22]. CM was harvested from CAF purified from patients undergoing surgery for colorectal cancer (termed CAF CM). For comparison with cancer cell activation, the CAF were treated with CM from human colon cancer cells, and the CM was then harvested (termed CAF CCM). CAF CM and CAF CCM were then treated to the murine myotubes (Fig. 1A).

Myotubes treated with CAF CCM showed atrophy, as shown by a significant reduction in myotube diameter (Fig. 1B, C). In contrast, CAF CM did not induce atrophy in the myotubes. CM from CCD-18Co normal human colon fibroblasts (termed NF CM) did not induce atrophy (Fig. 1B, C). Treatment with HCT116 CM did not produce myotube atrophy compared to CAF CCM (Supplementary Fig. 1B–D).

Myosin heavy chain (MYH2) is a major contractile protein in skeletal muscle. Western blotting showed that CAF CCM significantly reduced MYH2 expression (Fig. 1D, E). The E3 ubiquitin ligase, atrogin-1, is a key effector of muscle protein degradation and a biomarker for cancer cachexia [23]. CAF CCM treatment significantly upregulated atrogin-1 expression (Fig. 1D, E).

Cytokine Ab array analysis was used to identify secreted factors from cancer-activated CAF that induce myotube atrophy. Arrays for CAF CM, CAF CCM, and cancer cell CM were compared. The highest upregulated secreted factor in the CAF CCM was C-X-C motif chemokine 5 (CXCL5, also known as ENA-78) (Fig. 1F, G). Other upregulated factors included IL-6, stem cell factor (SCF) and vascular endothelial growth factor A (VEGF-A) (Fig. 1F, G). IL-6 is known to produce skeletal muscle wasting in cancer cachexia (reviewed in [24]). SCF is known to promote the survival of proliferation of multiple cell types [25]. VEGF has been shown to improve skeletal muscle regeneration [26]. Thus, SCF and VEGF-A were discounted from further analysis. The upregulation of CXCL5 in CAF CCM was confirmed using ELISA (Fig. 1H).

### CXCL5 neutralization prevents myotube atrophy induced by CAF CCM

**Fig. 2:**
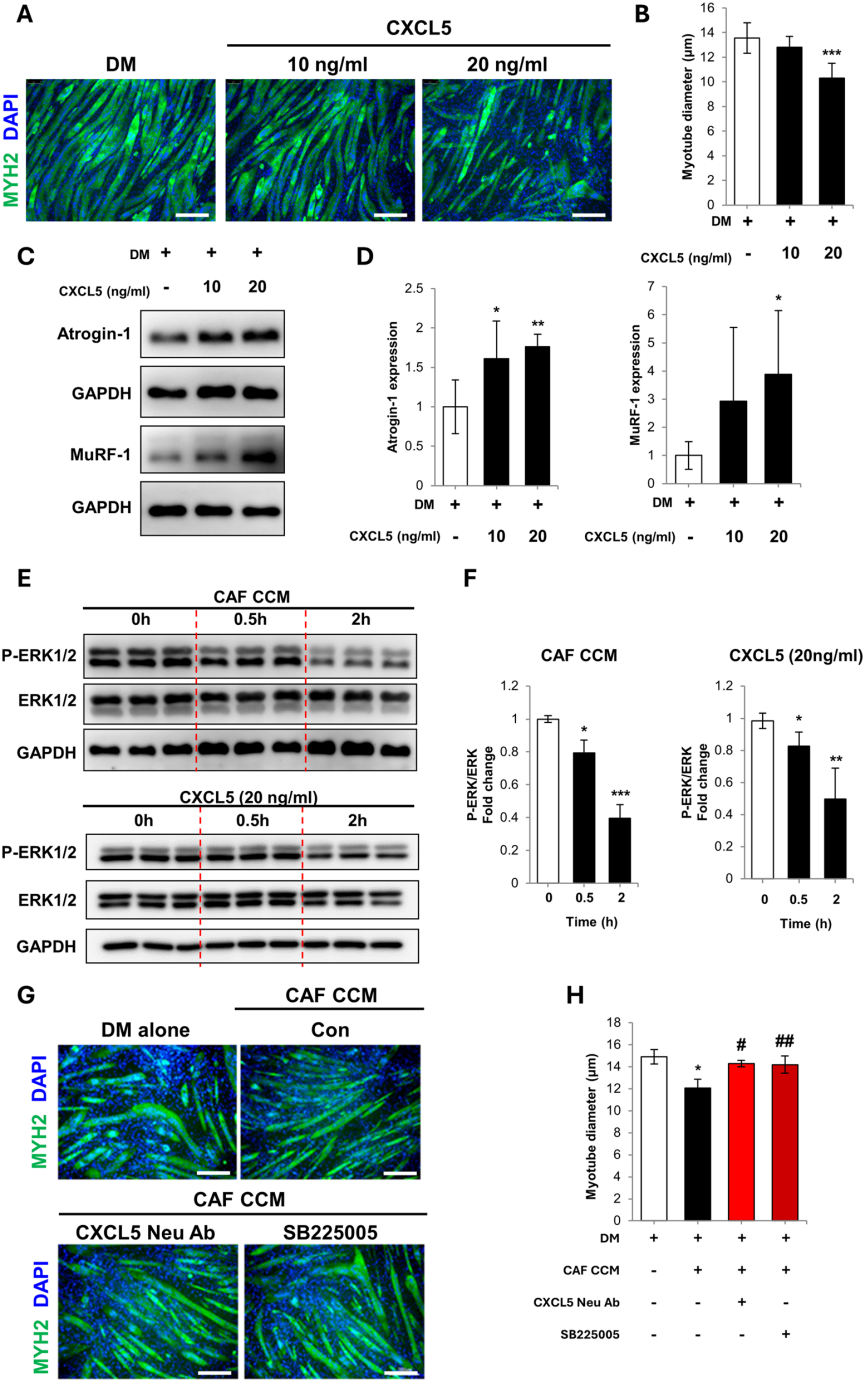
**A** Myosin heavy chain 2 (MYH2) immunostaining of C2C12 myotubes cultured in normal differentiation media (DM), DM plus 10 ng/mL CXCL5, or DM plus 20 ng/mL CXCL5 for 72 h. (scale bar = 150 μm) **B** Calculation of mean myotube diameter. **C** Western blot analysis of atrogin-1 and MuRF-1 expression in the treated myotubes. **D** Densitometry of atrogin-1 and MuRF-1 expression relative to GAPDH. **E** Western blot analysis of total extracellular signal-regulated kinase 1/2 (ERK1/2) and phosphorylated ERK1/2 in myotubes treated with CAF CCM or 20 ng/mL CXCL5 for 0.5 h and 2 h. **F** Densitometry of phosphorylated ERK1/2 relative to ERK1/2. **G** MHY2 immunostaining of C2C12 myotubes cultured for 72 h as follows: (1) Normal differentiation media (DM), (2) DM:CAF CCM (1:1) plus vehicle (0.1% DMSO), (3) DM:CAF CCM (1:1) plus 0.5 μg/mL CXCL5 neutralizing antibody, and (4) DM:CAF CCM (1:1) plus 22 nM SB225005 for 96 h (scale bar = 150 μm). **H** Calculation of mean myotube diameter. All experiments were conducted 3 times independently and the values are indicated as the mean ± SD. For **B**, **D**, **F** and **H**: * = *p* < 0.05, ** = p < 0.01 and *** = *p* < 0.001 indicate significantly increased compared to untreated. For **H**: # = *p* < 0.05 and ## = *p* < 0.01 indicates significantly increased compared to the DM:CCM (1:1) group

CXCL5 treatment alone induced myotube atrophy in a concentration dependent manner (Fig. 2A, B). Western blotting showed that CXCL5 increased the expression of atrogin-1 and MuRF-1 (Fig. 2C, D). Extracellular signal-regulated kinases (ERK) 1/2 signaling is required for skeletal muscle fiber maintenance and ERK1/2 inhibition has been shown to induce myofiber atrophy [27, 28]. CAF CCM treatment or CXCL5 treatment reduced ERK1/2 activity in the myotubes (Fig. 2E, F). The time points for checking ERK1/2 activity was based on a previous study by Mao, et al., that assessed CXCL5 induced ERK activation in gastric cancer epithelial-mesenchymal transition [29]. CXCL5 neutralization prevented CAF CCM-induced myotube atrophy (Fig. 2G, H). CXC motif chemokine receptor 2 (CXCR2) is the receptor for CXCL5. SB225005, a small molecule inhibitor of CXCR2, also prevented atrophy (Fig. 2G, H). CXCL5 neutralization reduced the upregulation of atrogin-1 and CXCR2 induced by CAF CCM treatment (Supplementary Fig. 2A, B). CXCR2 small molecule inhibition reduced the mean level of atrogin-1 and CXCR2 expression, but did not reach statistical significance.

IL-1α has been previously described as a cancer cell-derived factor that activates CAF [17]. The effect of IL-1α treatment on the expression of chemokines CXCL1, 2, 3, 5, 6, 7 and 8 in CAF was measured by qPCR. These chemokines were selected because they are known ligands for CXCR2 [30]. Among these chemokines, CXCL5 showed the strongest upregulation after IL-1α treatment (Supplementary Fig. 3A, B). IL-6, which has previously been linked to muscle wasting in cancer patients, was also upregulated, but at a lower level than CXCL5 (Supplementary Fig. 3B). NF-κB transcriptional activity has been shown to regulate CAF activation [31]. Western blot analysis showed that IL-1α treatment increased the phosphorylation of IκBα, which is required for NF-κB activation (Supplementary Fig. 3C, D). Accordingly, IL-1α treatment alone was sufficient to activate the CAF secretory phenotype and induce myotube atrophy (Supplementary Fig. 3E–G).

### CAF promote cancer cachexia in a humanized model

To assess the potential role of CAF in cancer cachexia, immunocompromised mice were xenografted with human colon cancer cells alone or cancer cells plus CAF. To ensure that the cancer cell transplantation alone does not produce cachexia, the time course and transplanted cell number were reduced compared to previous studies [32] (Supplementary Fig. 4). Using this approach, it was observed that cancer cell transplantation was insufficient to produce cachexia and can be used as a negative control (Supplementary Fig. 4A–E).

**Fig. 3:**
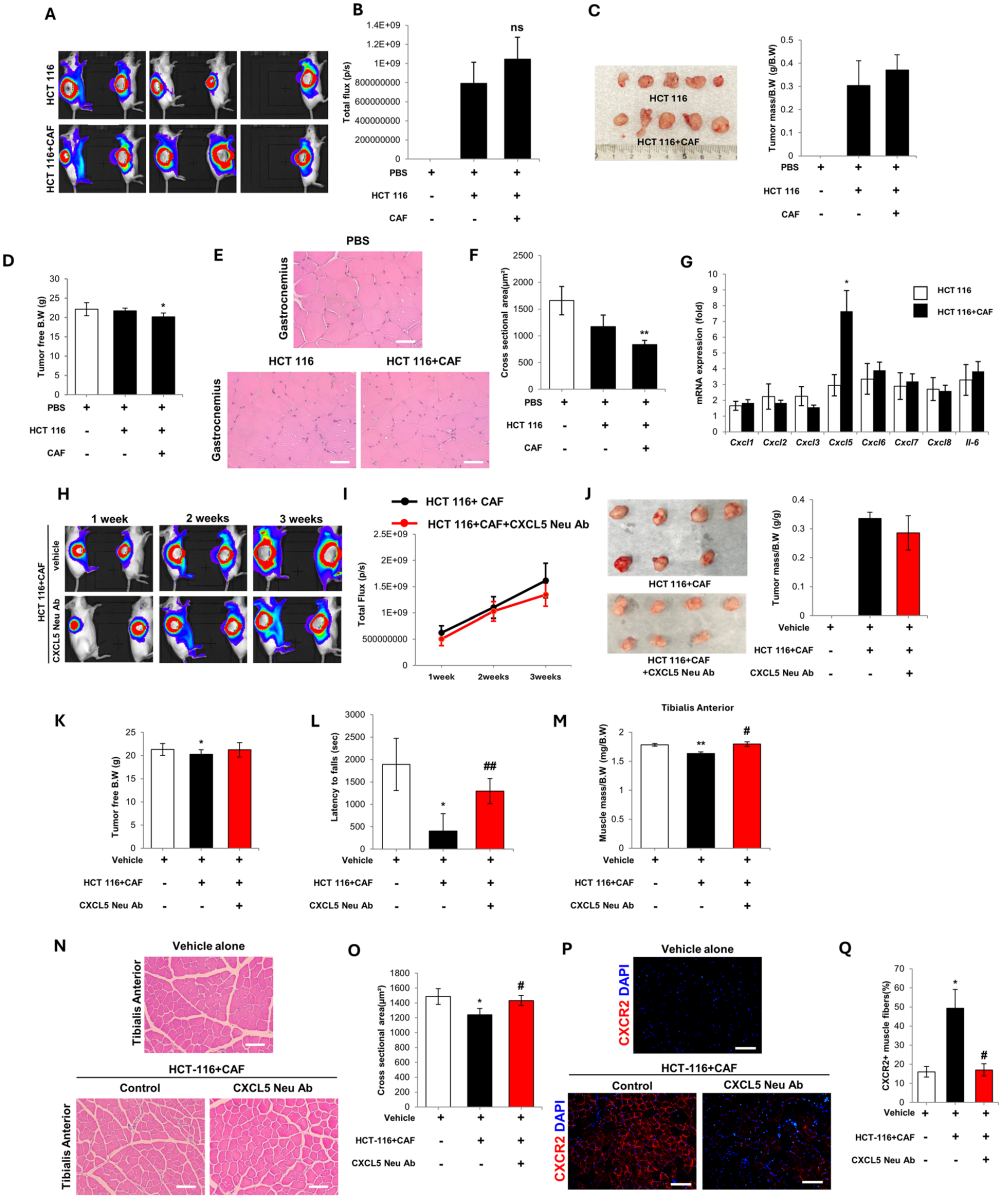
**A** IVIS imaging of NOD-SCID mice 3 weeks post-xenograft with 1 × 10^6^ human HCT 116 luc2 colon cancer cells, or 1 × 10^6^ HCT 116 luc2 cancer cells plus 2 × 10^6^ human colon CAF. **B** Mean total flux detected from the tumor at the end point (ns = not significant). **C** Dissected tumors and tumor mass at the 3 week endpoint. **D** Tumor free body weight (B.W) at the 3 week end point. **E** Representative images of H&E stained gastrocnemius muscle (scale bar = 50 μm). **F** Calculation of the myofiber cross sectional area. **G** qPCR analysis of CXCL1, 2, 3, 5, 6, 7, and 8 (known CXCR2 ligands) and IL-6 in the dissected tumor. **H** IVIS imaging of NOD-SCID mice at 3 weeks post-xenograft with human HCT 116 luc2 cancer cells plus human colon CAF, with or without CXCL5 neutralization (CXCL5 Neu Ab). **I** Mean total flux detected from the tumor at the 3 week end point. **J** Dissected tumors and tumor mass. **K** Tumor free body weight (B.W) at the 3 week end point. **L** Hanging tolerance in the treated mice. **M** Tibialis anterior (TA) muscle mass. **N** Representative H&E staining of the TA muscle (scale bar = 100 µm). **O** Calculation of the myofiber cross sectional area. **P** CXCR2 immunostaining of the TA muscle (scale bar = 100 µm). **Q** Proportion of CXCR2 positive myofibers. 5 mice per group were used for the experiments and the values are indicated as the mean ± SEM. For **D** and **F** * = *p* < 0.05 and ** = *p* < 0.01 indicate significantly decreased compared to PBS treated mice. For **G**, * = *p* < 0.05 indicates significantly increased compared to HCT 116 injected mice. For **I**–**Q** 5–7 mice per group were used for the experiments and the values are indicated as the mean ± SEM. * = *p* < 0.05 and ** = *p* < 0.01 indicate significantly decreased compared to vehicle alone. ^#^ = *p* < 0.05 and ^##^ = *p* < 0.01 indicate significantly increased compared to HCT 116 plus CAF

CAF co-transplantation with cancer cells did not affect tumorigenesis, because there was no significant effect on total flux or dissected tumor mass (Fig. 3A–C). In contrast, CAF co-transplantation significantly reduced tumor-free body weight (Fig. 3D). Histological analysis of the gastrocnemius muscle indicated that CAF co-transplantation induced myofiber atrophy, as shown by significantly reduced CSA (Fig. 3E, F; whole muscle image is shown in Supplementary Fig. 4F). qPCR analysis of the tumor tissue indicated that the presence of CAF during tumorigenesis upregulated the expression of CXCL5, whereas the expression of CXCL1, 2, 3, 6, 7, 8, and IL-6 were not significantly affected (Fig. 3G).

### CXCL5 neutralization ameliorates cancer cachexia

Immunodeficient mice were transplanted with human colon cancer cells plus CAF and treated with a CXCL5 neutralizing antibody. IVIS analysis showed that CXCL5 neutralization had no significant effect on tumor progression or tumor mass (Fig. 3H–J). CXCL5 neutralization prevented body weight loss (Fig. 3K). Neutralization improved muscle performance, as shown by increased latency to fall in the hanging tolerance test (Fig. 3L). Assessment of the TA muscle (composed primarily of fast twitch myofibers that are preferentially lost in cancer cachexia [33]) showed that neutralization increased muscle mass (Fig. 3M) and myofiber CSA (Fig. 3N, O; whole muscle image is shown in Supplementary Fig. 5G). Immunohistochemical analysis of the TA muscle showed that CXCR2 expression was upregulated in tumor bearing mice and downregulated by CXCL5 neutralization (Fig. 3P, Q).

To confirm that the CXCL5 neutralization antibody was effective at suppressing CAF-mediated cancer cachexia, the therapeutic effects were compared with a control IgG treatment group. It was observed that CXCL5 neutralization significantly reduced tumor signal flux at 3 weeks post-transplantation compared to control IgG (Supplementary Fig. 5A, B). However, there was no significant difference in tumor mass at the termination of the experiment, (Supplementary Fig. 5C, D). The CXCL5 neutralization antibody was shown to be effective at inhibiting CXCL5 detection by ELISA (Supplementary Fig. 5E). In addition, CXCL5 neutralization reduced CXCL5 levels in the serum of treated mice (Supplementary Fig. 5F).

CXCL5 neutralization increased the mean value of latency to fall in the hanging tolerance test, although this did not reach statistical significance (Supplementary Fig. 6A). Compared to the IgG control, CXCL5 neutralization significantly increased the mass of the quadriceps, gastrocnemius, and TA muscles (Supplementary Fig. 6B–D). Muscle fiber CSA may be considered as an indirect measure of myotube differentiation, because myotubes may contribute to the fiber CSA in cancer cachexia [34]. CXCL5 neutralization significantly increased CSA in the TA muscle (Supplementary Fig. 6E, F) and downregulated CXCR2 expression (Supplementary Fig. 6G, H). Western blot analysis demonstrated that CXCL5 neutralization effectively suppressed the expression of atrogin-1, MuRF-1, and CXCR2 in the TA muscle, whereas the IgG control used in the control group had no such effect (Supplementary Fig. 7A, B).

### CXCL5 neutralization upregulates myogenic PI3K-AKT-MyoG signaling and remodels the skeletal muscle ECM

**Fig. 4:**
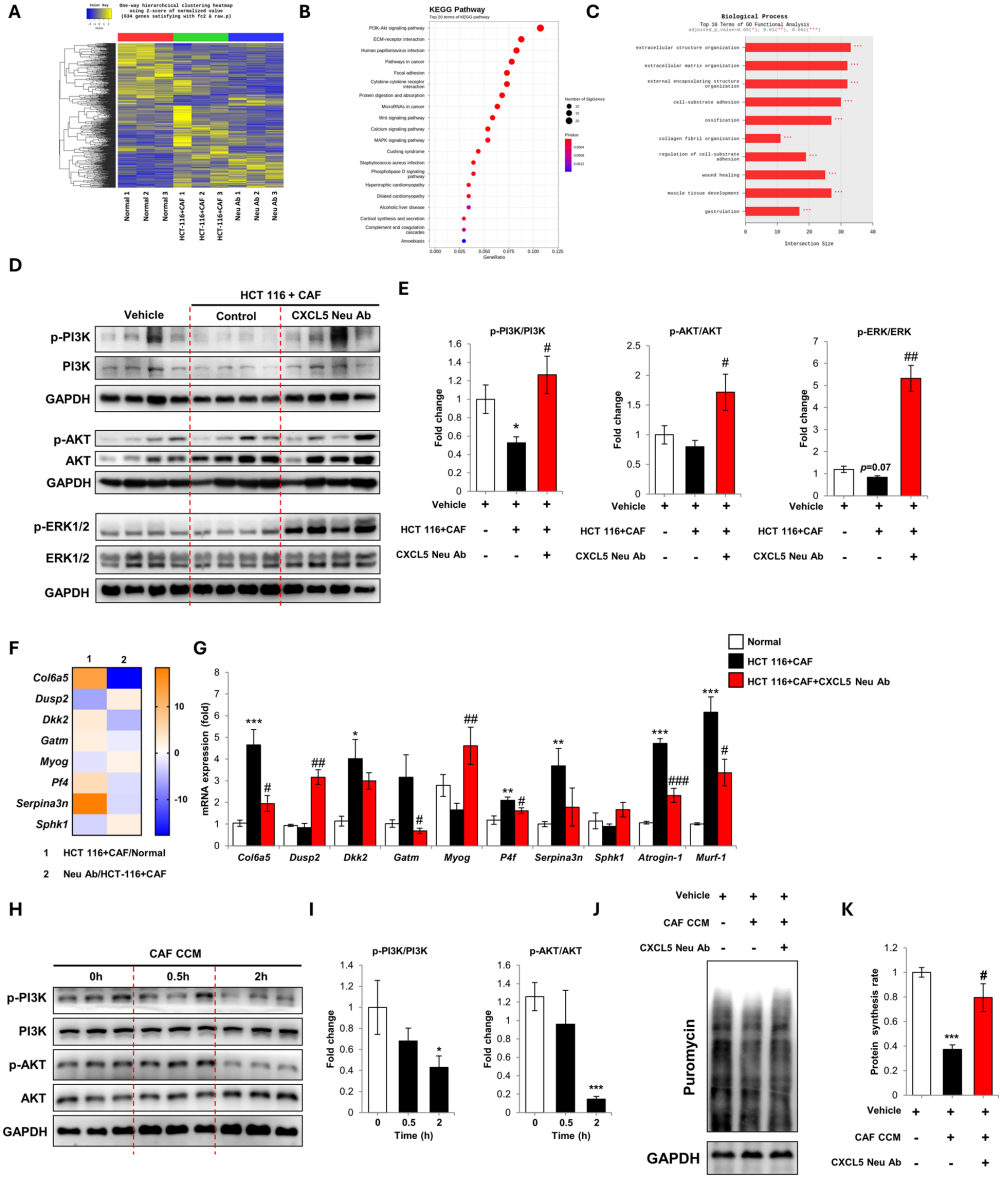
**A** Heat map of genes showing differential expression between the tibialis anterior (TA) muscles of NOD-SCID mice treated as follows: (1) No xenograft (Normal); (2) Xenograft with HCT 116 human cancer cells plus CAF (HCT 116 + CAF); (3) Xenograft with HCT 116 human cancer cells plus CAF, followed by treatment with a CXCL5 neutralizing antibody (Neu Ab). **B** KEGG (Kyoto Encyclopedia of Genes and Genomes) pathway analysis for the HCT-116 + CAF compared with Neu Ab treatment groups. **C** Gene ontology (GO) functional analysis for the HCT-116 + CAF group compared with Neu Ab treatment group. **D** Western blot analysis of PI3K-AKT and ERK1/2 phosphorylation in the dissected TA muscle from vehicle alone, HCT 116 + CAF, and Neu Ab-treated groups. **E** Densitometry of PI3K-AKT and ERK1/2 phosphorylation relative to PI3K-AKT and ERK1/2. **F** Heat map for genes showing differential expression between (1) HCT 116 + CAF xenograft compared to vehicle alone (HCT-116 + CAF/Normal), and (2) HCT 116 + CAF xenograft treated with a CXCL5 neutralizing antibody compared to HCT 116 + CAF xenograft alone (Neu Ab/HCT 116 + CAF). **G** qPCR analysis of the differentially expressed genes, in addition to atrogin-1 and MuRF-1 in the TA muscles. **H** Western blot analysis of PI3K-AKT phosphorylation in CAF CCM treated myotubes. **I** Densitometry of PI3K-AKT phosphorylation relative to PI3K-AKT. **J** Protein synthesis rate as determined by western blot analysis of puromycin incorporation (SUnSET assay). **K** Densitometry of puromycin incorporation normalized by GAPDH expression. For **E** and **G**: 4–5 mice per group were used for the experiments and the values are indicated as the mean ± SEM. * = *p* < 0.05, ** = *p* < 0.01 and *** = *p* < 0.001 indicate significantly increased or decreased compared to vehicle alone-injected mice (Normal). ^#^ = *p* < 0.05, ^##^ = *p* < 0.01 and ^###^ = *p* < 0.001 indicate significantly decreased compared to HCT 116 + CAF. For **I** and **K**: All values are indicated as the mean ± SD. * = *p* < 0.05 and *** = *p* < 0.001 indicate significantly decreased compared to vehicle alone. ^#^ = *p* < 0.05 indicates significantly increased compared to CAF CCM treatment

In vivo RNA seq of the TA muscle was used to identify the mechanisms by which CXCL5 neutralization prevents cancer cachexia. The heat map and volcano plots identified a total 540 differentially expressed genes (DEGS) between the neutralization and no treatment groups (Fig. 4A and Supplementary Fig. 8A). Kyoto Encyclopedia of Genes and Genomes (KEGG) pathway analysis showed that PI3K-AKT signaling was most affected by CXCL5 neutralization (Fig. 4B). Gene ontology functional analysis showed that biological processes related to extracellular matrix (ECM) structure and organization were primarily regulated by CXCL5 neutralization (Fig. 4C). Western blotting confirmed that CXCL5 neutralization upregulated the PI3K-AKT signaling pathway in skeletal muscle undergoing atrophy, with phosphorylation levels of both PI3K and AKT showing a significant increase (Fig. 4D, E). In addition, CXCL5 neutralization increased phosphorylated ERK levels in the TA muscle (Fig. 4D, E).

DEGs reported to be linked to ECM remodeling or skeletal muscle wasting included collagen type VI alpha 1 chain (Col6a), dual specificity phosphatase 2 (Dusp2), Dickkopf WNT signaling pathway inhibitor 2 (Dkk2), glycine amidinotransferase (Gatm), platelet factor 4 (P4f), serpin family A member 3 (serpina3n), sphingosine kinase 1 (Sphk1), and myogenin (MyoG) (Fig. 4F and Supplementary Fig. 8B). The transcription factor MyoG is a master regulator of myogenesis and essential for muscle development (reviewed in [35]) and has previously been shown to be downregulated in cancer cachexia [36]. qPCR analysis also confirmed the downregulation of Col6a, Gatm and P4f, and the upregulation of Dusp2 (Fig. 4G). In line with the (KEGG) pathway analysis, western blotting showed that CCM treatment reduced PI3K and AKT phosphorylation (Fig. 4H, I). CXCL5 treatment alone also reduced AKT phosphorylation in C2C12 myotubes (Supplementary Fig. 8C, D). PI3K-AKT signaling is known to regulate the protein synthesis rate in skeletal muscle. Accordingly, the SUnSET assay confirmed that CXCL5 neutralization increased protein synthesis (Fig. 4J, K).

### CXCL5 neutralization ameliorates skeletal muscle wasting induced by CXCL5 and IL-6

The cytokine array data showed that the known cachexokine IL-6 was also upregulated in the CAF CCM treated myotubes. ELISA confirmed the increase of IL-6 in CAF CCM (Supplementary Fig. 9A) and IL-6 treatment alone was sufficient to induce myotube atrophy (Supplementary Fig. 9B, C). To examine any possible synergic effect of CXCL5 and IL-6, lowered concentrations were treated to the myotubes (1 ng/mL CXCL5 and 2 ng/mL IL-6). CXCL5 and IL-6 co-treatment at the lower concentrations significantly decreased myotube diameter, reduced MYH2 levels, and upregulated atrogin-1 expression (Supplementary Fig. 9D–G). Co-treatment was also effective at reducing levels of phosphorylated ERK and AKT in the myotubes (Supplementary Fig. 9H, I). Therefore, to assess the effect of neutralization on CXCL5-induced atrophy in vivo, IL-6 used for co-treatment with CXCL5.

**Fig. 5:**
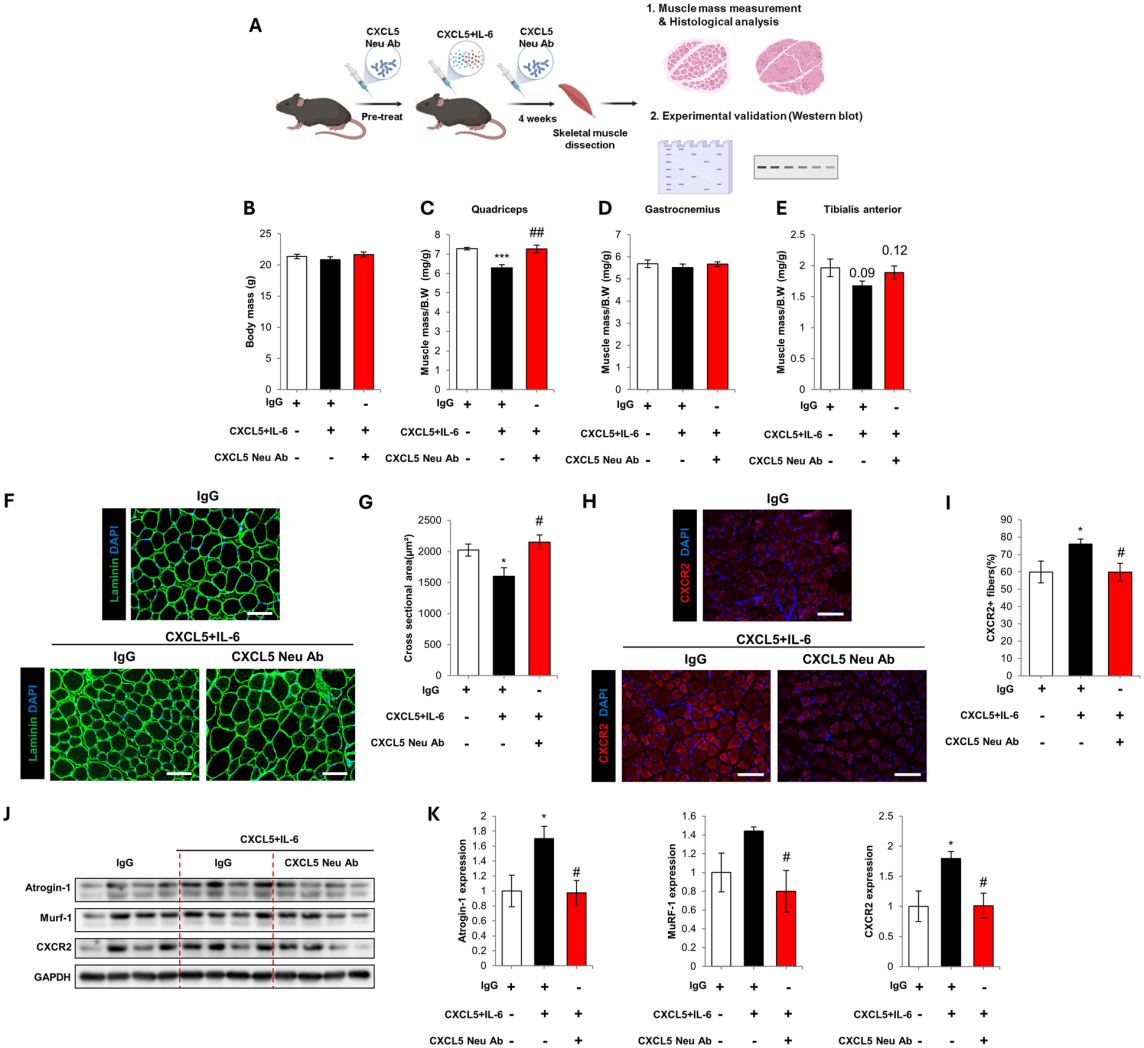
**A** Schematic of the protocol to investigate CXCL5 neutralization in a model of cytokine-induced skeletal muscle wasting. C57BL6/J mice were treated with 40 ng/kg CXCL5 and 80 ng/kg IL-6 for 4 weeks with or without 120 μg/kg CXCL5 neutralizing antibody. 120 μg/kg IgG1 was used as control. **B** Body weight at the end point of experiment. **C** Quadriceps muscle mass. **D** Gastrocnemius muscle mass. **E** Tibialis anterior (TA) muscle mass. **F** Laminin staining of the TA muscle (scale bar = 150 µm). **G** TA myofiber cross sectional area. **H** CXCR2 staining of the TA muscle (scale bar = 150 µm). **I** The proportion of CXCR2 positive fibers in TA muscle. **J** Western blot analysis of atrogin-1, MuRF-1 and CXCR2 expression in the TA muscle. **K** Densitometry of atrogin-1, MuRF-1 and CXCR2 expression normalized by the expression of GAPDH. For **B**–**I,** 7 mice per group were used for the experiments and for **J**–**K,** 4 mice per group were used for the experiments and the analysis was carried out two times. The values were indicated as the mean ± SEM. * = *p* < 0.05 and ** = *p* < 0.01 indicate significantly increased or decreased compared to IgG1 control. ^#^ = *p* < 0.05 and ^##^ = *p* < 0.01 indicate significantly decreased compared to CXCL5 + IL-6 + IgG1

C57BL/6 mice were treated with 40 ng/kg CXCL5 and 80 ng/kg IL-6 three times per week for 4 weeks. This dosing regimen was calculated as follows: CXCL5 was measured as 1 ng/mL and IL-6 at 2 ng/mL in the CAF CCM by ELISA. Assuming a mouse weight of 25 g and a predicted blood volume is 1 mL, this corresponds to a CXCL5 dose of 40 ng/kg and IL-6 dose of 80 ng/kg for the animal model study. The selection of 4 weeks’ as the treatment time was based on a pilot study of CXCL5 and IL-6 treatment in the mice. One experimental group was treated with a CXCL5 neutralizing antibody or an IgG control (Fig. 5A). Cytokine treatment in the presence or absence of CXCL5 neutralization did not significantly affect body weight (Fig. 5B). Quadriceps muscle mass was significantly reduced by cytokine treatment (Fig. 5C). CXCL5 neutralization increased the mean muscle mass values of the gastrocnemius and TA muscles, but this did not reach statistical significance (Fig. 5D, E). Immunohistochemical analysis of the TA showed that myofiber CSA was reduced by cytokine treatment and increased by neutralization (Fig. 5F, G). In addition, cytokine treatment upregulated CXCR2 expression in the TA muscle, which was normalized by neutralization (Fig. 5H, I). Western blotting analysis indicated that cytokine treatment increased atrogin-1, MuRF-1 and CXCR2 levels in the TA. Neutralization inhibited this rise in atrogin-1, MuRF-1, and CXCR2 levels (Fig. 5J, K). qPCR analysis confirmed that neutralization suppressed atrogin-1 and MuRF-1 upregulation, and increased MyoG expression, compared to the IgG control (Supplementary Fig. 10).

### CXCL5 neutralization prevents CAF CCM-induced atrophy in human primary skeletal muscle cells

**Fig. 6:**
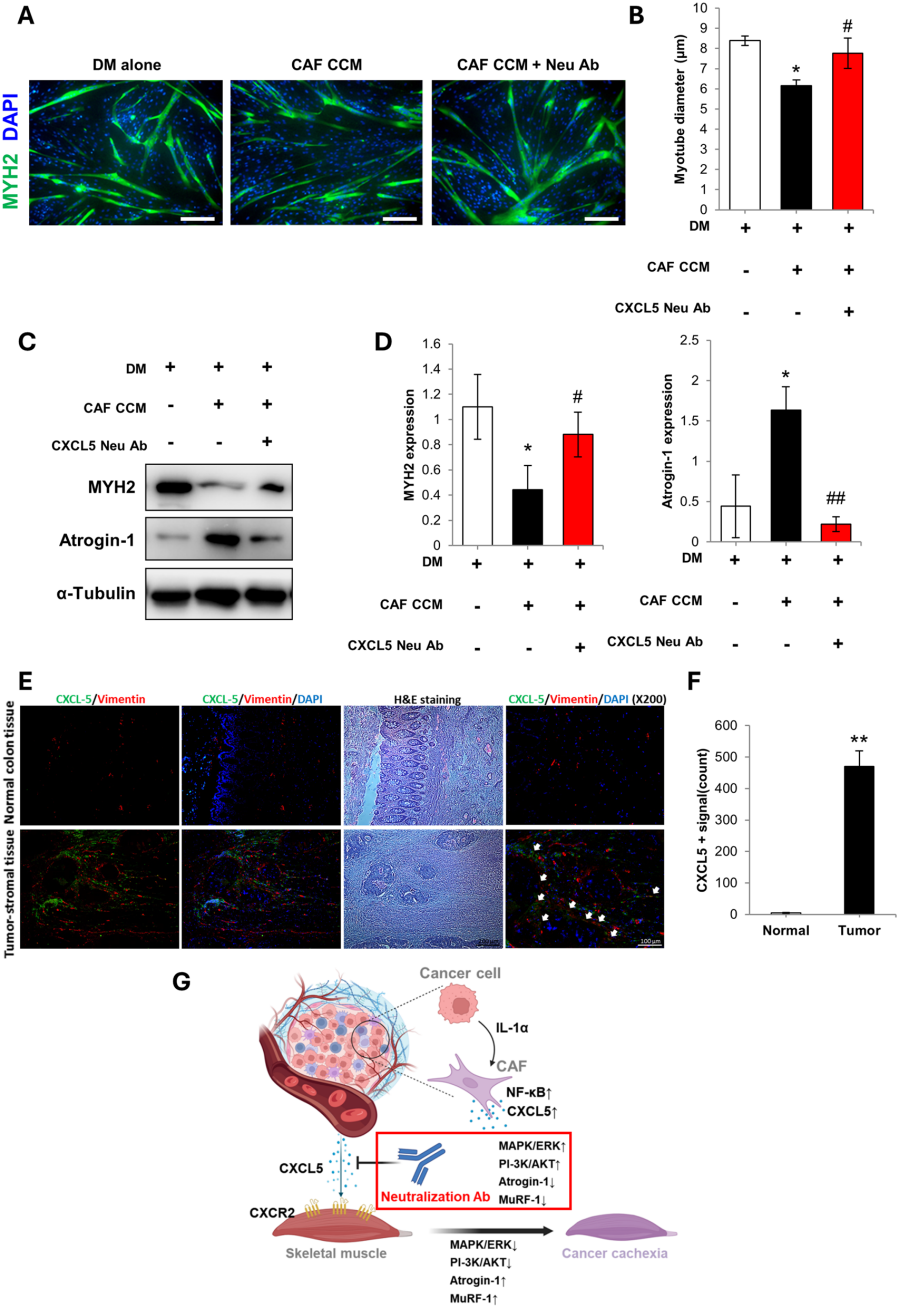
**A** Representative MYH2-stained images of human donor myotubes cultured as follows: (1) Differentiation media (DM) for 72 h; (2) Treatment with human HCT 116 colon cancer cell-stimulated CAF CM (CAF CCM) for 72 h; (3) Treatment with CAF CCM and CXCL5 neutralizing antibody (CAF CCM + Neu Ab) for 72 h (scale bar = 100 μm). **B** Calculation of mean myotube diameter. **C** Western blot analysis of MYH2 and atrogin-1 expression. **D** Densitometry of MYH2 and atrogin-1 expression relative to α–tubulin. **E** Immunohistochemical analysis of CXCL5 and vimentin expression in tumor-stromal and normal tissues obtained from a colon carcinoma patient. White arrows indicate overlapping CXCL5 and vimentin immunostaining. Quantification of CXCL5 fluorescence in the tumor-stromal tissues is also shown (** = *p* < 0.01 compared to normal). All experiments were conducted 3 times independently and the values were indicated as the mean ± SD. For **B** and **D**: * = *p* < 0.05 indicate significantly increased or decreased compared to DM alone. ^#^ = *p* < 0.05 and ^##^ = *p* < 0.01 indicate significantly increased or decreased compared to CAF CCM. **G** Working model of the role of CAF in cancer cachexia progression. Molecular crosstalk between cancer cells and CAF in the tumor microenvironment induces the secretion of chemokine CXCL5 by CAF. CXCL5 activates muscle atrophy signaling, as shown by decreased PI3K-AKT and ERK phosphorylation and upregulation of the key atrogenes, atrogin-1 and MuRF-1, causing the skeletal muscle loss observed in cancer cachexia

Human primary skeletal muscle myotubes underwent atrophy when treated with CAF CCM, as shown by reduced myotube diameter. CXCL5 neutralization prevented atrophy in the CAF CCM treated human myotubes (Fig. 6A, B). Western blotting showed that CAF CCM treatment reduced MYH2 and increased atrogin-1 expression. CXCL5 neutralization normalized MYH2 and atrogin-1 expression (Fig. 6C, D).

The occurrence and distribution of CXCL5 expression in human tumors was investigated in samples obtained from patients that underwent tumor resection surgery for colon cancer. Immunostaining showed that CXCL5 was preferentially expressed in the CAF-rich stromal compartment of the tumor, with vimentin displaying an overlapping pattern of expression with the stroma (Fig. 6E, F and Supplementary Fig. 11). In contrast, normal colon tissue was relatively devoid of CXCL5 staining. This overlapping expression of CXCL5 within the vimentin positive stroma was consistently observed in the patient tumor tissues (Supplementary Fig. 11).

## Discussion

This study investigated the potential role and underlying molecular mechanism of non-cancer cell populations in tumors on the development of cachexia. CAF are a major cell population of the tumor microenvironment and it was discovered that inducible CXCL5 secretion from CAF contributes to cachexia, which can be mitigated by CXCL5 neutralization. Intercellular ‘crosstalk’ between different cell types within the tumor microenvironment has been shown to regulate cancer progression, metastasis, and response to therapy [16]. The results presented herein show a new aspect of this crosstalk: the promotion of cancer cachexia by cancer cell-stimulated CAF.

Conditioned media from cancer-stimulated CAF (CAF CCM) was effective at inducing myotube atrophy in vitro, as shown by reduced myotube diameter, upregulated atrogin-1, and lower MYH2 levels. In contrast, conditioned media from non-stimulated CAF (CAF CM) had no significant effect on these parameters. This result validates the requirement for cancer cell crosstalk via soluble factors to induce myotube atrophy. Previous studies have shown that atrogin-1 induction can be considered as the primary event during cancer cachexia, and this occurs before the appearance of cachexia [23]. Previous studies has identified cancer cell factors that stimulate CAF in other disease contexts. For example, IL-1α secreted from oral carcinoma cells (OSCC) was shown to induce chemokine (C–C motif) ligand 7 (CCL7) release from oral CAF, which in turn promoted OSCC invasion [17]. In this study, colon cancer cell-derived IL-1α was also confirmed as the secreted factor that induced the activation of colon CAF. A variation in the degree of myotube differentiation was observed in the DM group of Figs. 1B, 2A, and 2G. This may be due to a number of reasons. For example, our own observations indicate that batch variation in the supply of culture serum can influence the differentiation efficiency of the myoblasts. In addition, passage number is also known to influence C2C12 differentiation.

A CXCL5 neutralizing antibody or small molecule antagonist of CXCR2 (the cognate receptor for CXCL5) prevented myotube atrophy induced by treatment with CAF CCM, as shown by reduced myotube diameter. CXCR2 receptor is known to be expressed in myoblasts and skeletal muscle [37]. In addition, skeletal muscle CXCR2 expression is upregulated in a model of cancer cachexia [38]. CXCR2 is also a receptor for IL-8 and this cytokine has also been linked to cancer cachexia [39]. Therefore, to test the effects of CXCL5 inhibition, it would be preferable to specifically target the chemokine rather than the multiligand receptor. It is interesting to note that there were some differences in the effects of CXCL5 neutralization or small molecule inhibition of CXCR2. Small molecule treatment did not reach statistical significance for atrogin-1 and CXCR2 downregulation, although the mean value was reduced, suggesting that CXCL5 neutralization is more effective at preventing myotube atrophy-related mechanisms.

CXCR2 is a G-protein coupled receptor that triggers the mobilization of intracellular calcium ions. This in turn activates phospholipase D (PLD) and the MAPK pathway [40]. There have been few reports concerning PLD activation in skeletal muscle atrophy. One previous study showed that PLD activation induces hypertrophy in normal muscle, whereas the PLD2 isoform inhibited muscle recovery during unloading [41]. Moreover, cellular levels of the PLD substrate phosphatidylcholine are reduced in denervated or dystrophic muscles [42]. Blocking PLD activity as a therapeutic strategy for cancer cachexia may be a promising area for future research.

In the humanized model of cancer cachexia, CAF co-transplantation with cancer cells produced a greater loss of skeletal muscle mass compared to cancer cell transplantation alone. CXCL5 neutralization was also shown to increase skeletal muscle mass. Recently, there has been greater appreciation that maintaining muscle mass may be more important than function when assessing therapeutic approaches for cancer cachexia [43]. This is in contrast to other types of muscle atrophy, such as sarcopenia, where improvements in function take priority. Increasing muscle mass in cancer cachexia is thought to allow the patient to ‘buy more time’ for tumor intensive therapies before wasting reaches a critical state [43].

In this study female mice were used for both the humanized model and cytokine treatment model of cancer cachexia. It should be noted that female patients and mice are reported to be more resistant to the development of cancer cachexia compared to males, although the mechanisms responsible for this difference are unclear [44, 45]. Thus, the degree of cachexia observed in the humanized model and cytokine treatment model may be more severe if male mice were selected. The therapeutic effect of CXCL5 neutralization on cancer cachexia may depend on whether CXCR2 signaling contributes to these differences in cachexia progression observed in males and females. This should be an interesting subject for further investigation. The patients’ cancer tissues analyzed in this study all showed elevated CXCL5 immunostaining in the stromal region. A previous report demonstrated increased CXCL5 levels in males with pancreatic cancer [46], although the expression pattern within the tumors was not determined.

Female mice were also used for this study because they were more suitable for subcutaneous tumor xenografts. Male mice tended to be more aggressive and could injure the developing tumors in other mice. Some male mice were observed to kill weaker mice with more advanced tumor burden. Pooling was also easier for the female mice due to less aggression. Based on previous studies, female mice were found to yield more consistent results in tumor studies and the gender of SCID mice did not have a significant impact on tumor development or drug responses [47, 48]. Data variability in female mice was reported to be more influenced by the circadian cycle rather than estrogen levels [49]. It has also been shown that HCT116 xenografts into female mice can be utilized to study tumor biology (for example [50, 51]). Our survey of the research literature regarding the role of the estrous cycle in cachexia development found reports showing a preventative effect (e.g. Counts, et al. [52]) or a contributing factor (e.g. Hetzler, et al. [53]). For the model of colon cancer induced cachexia, we were guided by the results from Banh, et al., who reported that cachexia progression was more dependent on tumor and adipose mass, rather than gender or ovariectomy status [54]. However, further studies in male mice would be recommended for validating the role of CXCL5 in cancer cachexia and can better match hormonal regulation.

In the cytokine treatment model, only the quadriceps muscle showed a significant reduction of muscle mass and recovery with CXCL5 neutralization. This quadriceps-specific effects may be due to the type of muscle atrophy model used in this part of the study. When cancer cells plus CAF were transplanted into mice, the muscle atrophy effect was stronger, with a significant reduction in quadriceps, gastrocnemius and TA muscle mass. As the quadriceps is the largest muscle analyzed, it may be easier to detect changes in mass. A change in quadriceps mass will represent a larger proportion of the total mass, compared to other muscles, making it more detectable.

RNA-Seq showed that CXCL5 neutralization modulates the skeletal muscle ECM. ECM disarrangement has been observed in models of cancer cachexia and ECM gene signatures are an important indicator of muscle hypertrophic responses to resistance exercise [55]. KEGG pathway analysis revealed that PI3K-AKT signaling was the major signaling module upregulated by neutralization. PI3K-AKT signaling is a major regulator of muscle hypertrophy via activation of mammalian target of rapamycin (mTOR) and inhibition of FoxO3a [56, 57].

PI3K-AKT signaling also prevents muscle atrophy by suppressing NF-κΒ activity, which a main effector pathway for cytokine-mediated muscle degradation [56, 57]. However, in non-muscle tissues, PI3K/AKT inhibition has been shown to suppress NF-κΒ activity [58, 59]. In addition, NF-κB is known to produce anti-apoptotic effects, whereas in skeletal muscle NF-κB activity is an important activator for myofiber atrophy [60].

These differences may be due to how NF-κΒ activity is regulated in skeletal muscle. For example, in fasting skeletal muscle, NF-κΒ activity is upregulated and AKT signaling is downregulated [60]. This is due to regulation by the protein acetylase, general control non-depressible 5 (GCN5). Knockdown of GCN5 decreased NF-κΒ p65K310 acetylation and activity in fasting muscle, and also upregulated AKT signaling. An alternative mechanism to activate NF-κΒ in muscle atrophy is via the upregulation of FOXO3, which is a master regulator of atrophy signaling. FOXO3 has been shown to activate NF-κΒ in conditions of cell stress [61]. Consequently, PI3K/AKT signaling, which is known to inhibit FOXO3, may indirectly downregulate NF-κΒ activity by targeting this master gene in skeletal muscle.

Upregulated genes linked to muscle tissue development included MyoG (myogenin). MyoG is a member of the MyoD family of transcription factors and master regulator of myogenesis [62]. Ectopic MyoG expression alone is sufficient to switch on the myogenic gene expression program in non-muscle cells [63]. MyoG is also an essential regulator of adult myofiber growth and muscle stem cell homeostasis [64]. The PI3K-AKT pathway is known to be an upstream regulator of MyoG expression in muscle cells [65]. It can be envisaged that this pathway is also responsible for the therapeutic effect of CXCL5 neutralization, although further experimental evidence would be required to show the connection between PI3K-AKT signaling and MyoG upregulation in this disease context. The TA was the primary muscle type used in this analysis, because it is composed primarily of fast twitch myofibers that are preferentially lost in cancer cachexia [33, 66]. In addition, the TA muscle is commonly used for studies of muscle atrophy [67]. Although other muscle types were assessed in the current study (gastrocenemius and quadriceps, which is a complex of individual muscles: Rectus Femoris, Vastus Lateralis, Vastus Medialis, Vastus Intermedius), additional muscles should be analyzed in more detail to further characterize the role of CXCL5 in cancer cachexia progression.

Patient colon tumor tissue immunostaining revealed that areas of CXCL5 expression occur within the CAF-rich stromal compartment, as shown by colocalization with vimentin. The ability of CAF to secrete CXCL5 has been previously reported [68]. Numerous previous studies have linked CXCL5 expression to reduced survival in cancer patients. For example, a meta-analysis of multiple cancer types showed that elevated CXCL5 levels was associated with lower overall survival, progression-free survival and recurrence-free survival [69]. Analysis of online databases and tissue microarray staining in pancreatic ductal adenocarcinoma patients showed that elevated CXCL5 is a marker of poor prognosis [70]. In addition, cytokine array and ELISA analysis of serum from cancer patients and healthy volunteers found that CXCL5 levels are a prognostic marker for colorectal cancer [71]. The results in the current study suggest that the influence of CXCL5 levels on cancer patient survival may, at least in part, be due to an effect on the progression of cachexia. Future studies could employ bioinformatics-based analyses of clinical data to delineate the relationship between CXCL5 levels and the severity of cachexia in cancer patients.

Although CAF are a major non-cancer cell component of tumors, other cell types are also found in the microenvironment, such as immune cells (including tumor-associated macrophages (TAMs) and tumor-infiltrating neutrophils (TINs)) and cancer-associated adipocytes (CAAs) [16]. The potential roles of these cell populations in the progression of cachexia are relatively unexplored compared to cancer cells. It may be rewarding to characterize the cancer cell-inducible factors in these cell types and test their effect on myotube atrophy.

There have been numerous studies of CXCL5 expression in developing tumors. CXCL5 have been described as a ‘coachman’ that drives cancer progression via immunosuppression, angiogenesis and metastasis [72]. Elevated CXCL5 levels in patients were shown to correlate with survival time and recurrence [73]. In melanoma, CAF secreted CXCL5 and upregulated programmed cell death protein 1 (PD-L1) expression in cancer cells [68, 73]. CXCL5 has also been studied as a therapeutic target for the prevention of tumor angiogenesis or as an enhancer of cancer drug efficacy [74, 75]. The results presented in this study reveal a new aspect of CAF-derived CXCL5 secretion in the progression of cancer cachexia. Thus, elevated CXCL5 levels in patients may be associated with increased weight loss and/or a more rapid progression of cachexia, especially if CXCL5 is upregulated in the tumor stromal compartment. This clinical aspect should be an interesting subject for further investigation. Moreover, drug development strategies targeting CXCL5 in cancer, such as small-interfering RNAs, small molecules, or antibodies, may focus on cachexia prevention as a therapeutic readout in future clinical trials.

In conclusion, these results show that CAF promote cachexia via cancer cell-inducible CXCL5 secretion. We postulate that the role of CAF-secreted CXCL5 in cancer cachexia is due to activation of the CXCL5 receptor CXCR2 in skeletal muscle. Cancer cells stimulate CXCL5 secretion from CAF, which enters the bloodstream and binds to the skeletal muscle receptor to activate pro-cachexia signaling pathways. The neutralizing antibody binds CXCL5 in the serum and prevents its binding to the receptor on muscle tissue, which inhibits pro-cachexia signaling and maintains muscle mass. Our results show that CXCL5 treatment can induce myotube atrophy and CXCR2 expression is upregulated in the skeletal muscle of mice transplanted with cancer cells plus CAF. This mechanism may function without the requirement for immune cells. The CXCL5 neutralizing antibody treatment had no significant effect on tumor growth (Fig. 3H-J). Therefore, we believe that the neutralizing antibody targets muscle tissue, rather than the developing tumor. The neutralizing antibody treatment protects skeletal muscle mass via the upregulation of PI3K-AKT signaling and downstream MyoG activity. To our knowledge, this study is the first mechanistic investigation of the role of CAF in cancer cachexia progression. In addition to a direct effect on skeletal muscle wasting, cancer cells also contribute to cachexia by regulating the secretome of CAF (Fig. 6G). Thus, CAF can be viewed as the ‘henchmen’ of cancer cells in cachexia progression, providing muscle-derived amino acids that are absorbed by the cancer cells and accelerate tumor growth and metastasis [5, 76]. For example, glutamine released from atrophied muscles is converted to glutamate within the tumor and promotes metastasis [77]. Overall, these results can increase our understanding of the cellular interactions that regulate the molecular effectors of cancer cachexia and offer a novel therapeutic strategy for maintaining skeletal muscle mass and promoting longevity in patients with advanced cancer.

## Conclusions

CAF are known to have major roles in tumorigenesis, immune evasion, and metastasis. The results of this study show that CAF are also potent inducers of cancer cachexia, a devastating wasting syndrome that impedes therapy effectiveness and accounts for 20–30% of cancer-related mortality. This was found to occur via a triple crosstalk among CAF, cancer cells, and skeletal muscle: cancer cells stimulate CAF to upregulate secretion of the chemokine CXCL5, which enters the circulation, binds to CXCR2 on skeletal muscle, and activates atrophy-related signaling pathways that drive muscle wasting. Neutralization of CXCL5 markedly attenuated cachexia development in animal models, including a human colon carcinoma xenograft. These findings identify CXCL5 blockade as a promising therapeutic strategy, warranting evaluation across multiple cancer types and progression toward clinical translation.

## Availability and data materials

The data that support the findings of this study are available from the corresponding authors on request.

## Supplementary Information

Additional file 1: Figure 1. A) Immunostaining of vimentin (a fibroblast marker), and cytokeratin (an epithelial marker) in CCD-18Co normal colon fibroblasts (NF), colon carcinoma CAF purified from 2 patients (CAF-HS2 and CAF HS3), and HCT 116 cancer cells. B) Myosin heavy chain 2 (MYH2) immunostaining of C2C12 myotubes cultured in normal myotube differentiation media (DM) and treated with DM, HCT116 or CAF CCM for 72 h (scale bar=150 μm). C) Calculation of mean myotube diameter. D) Myotube diameter distribution (μm). *=p<0.05, **=p<0.01.

Additional file 2: Figure 2: A) Western blot analysis of atrogin-1 and CXCR2 expression in C2C12 myotubes cultured in normal differentiation media with 0.1% DMSO (vehicle), CAF CCM and CAF CCM plus CXCL5 neutralizing antibody (0.5 μg/mL), or SB225005 (22 nM), an inhibitor of CXCR2, for 72h. α-Tubulin was used as a loading control. B) Densitometry of atrogin-1 and CXCR2 expression relative to α-tubulin. All experiments were performed 3 times independently and the values were indicated as the mean ± SD. *=p<0.05 indicate significantly increased compared to vehicle treated. #=p<0.05 indicate significantly decreased compared to CAF CCM treated.

Additional file 3. Figure 3: A) Schematic diagram of CAF activation by IL-1α treatment. B) qPCR analysis of CXCL1, 2, 3, 5, 6, 7, and 8, and IL-6 expression in IL-1α (1 ng/mL) treated CAF compared to CAF alone in serum free media with vehicle (0.1% BSA in PBS). C) Western blot analysis of IκBα phosphorylation and NF-κB expression. D) Densitometry of IκBα phosphorylation and NF-κB expression relative to IκB and GAPDH, respectively. E) Schematic diagram of induction of myotube atrophy by IL-1α-treated CAF. CAF CM was collected after IL-1α treatment (1 ng/mL) and incubated with C2C12 myotubes for 72 h. F) Representative MYH2-stained images of C2C12 myotubes cultured as follows: (1) Differentiation media (DM); (2) Treatment with non-stimulated CAF CM; (3) Treatment with IL-1α-stimulated CAF CM (scale bar = 100 μm). G) Calculation of mean myotube diameter. All experiments were performed 3 times independently and the values were indicated as the mean ± SD. ***= p<0.001 indicates significantly decreased compared to the non-stimulated CAF or DM alone group.

Additional file 4. Figure 4: A) IVIS imaging of NOD-SCID mice 3 weeks post-xenograft with 1x106 or 2x106 HCT 116 cancer cells. Mean total flux detected from the tumor at the end point is also shown. B) Body weight changes for 3 weeks post-xenograft with 1x106 or 2x106 HCT 116 cancer cells. C-E) Gastrocnemius, soleus and TA muscle mass in vehicle alone-treated NOD-SCID (designated as ‘vehicle’) and NOD-SCID mice 3 weeks post-xenograft with 1x106 or 2x106 HCT 116 cancer cells. F) Low magnification image of the whole gastrocnemius muscle of NOD-SCID mice 3 weeks post-xenograft with 1x106 human HCT 116 luc2 colon cancer cells, or 1x106 HCT 116 luc2 cancer cells plus 2x106 human colon CAF (scale bar=1 mm). 3 mice per group were used for the experiments and the values were indicated as the mean ± SEM.

Additional file 5. Figure 5: A) IVIS imaging of NOD-SCID mice at 3 weeks post-xenograft with human HCT 116 luc2 cancer cells plus human colon CAF, with or without CXCL5 neutralization (CXCL5 Neu Ab) and 120 μg/kg IgG1 used as control. B) Mean total flux detected from the tumor at the 3 week end point. C-D) Dissected tumors and tumor mass. E) CXCL5 neutralization was tested with CXCL5 ELISA results on cell free condition. F) CXCL5 concentration in the collected serum. G) Low magnification image of the whole tibialis anterior muscle of NOD-SCID mice 3 weeks post-xenograft with human HCT 116 luc2 cancer cells plus human colon CAF, with or without CXCL5 neutralization (CXCL5 Neu Ab) (scale bar=1 mm). For A-D and F), 7 mice per group were used for the experiments and the values were indicated as the mean ± SEM. *=p<0.05 and **=p<0.01 indicate significantly increased or decreased compared to IgG1 control. #=p<0.05 and ##=p<0.01 indicate significantly decreased compared to HCT116 plus CAF+IgG1.

Additional file 6. Figure 6: A) Hanging tolerance in the treated mice. B) Quadriceps muscle mass. C) Gastrocnemius muscle mass. D) Tibialis anterior (TA) muscle mass. E) Laminin staining of the TA muscle (scale bar=150 µm). F) TA myofiber cross sectional area. G) CXCR2 staining of the TA muscle (scale bar=150 µm). H) The proportion of CXCR2 positive fibers in TA muscle. 7 mice per group were used for the experiments and the values are indicated as the mean ± SEM. *=p<0.05 and **=p<0.01 indicate significantly decreased compared to IgG1 control. #=p<0.05 and ##=p<0.01 indicate significantly increased compared to HCT 116 plus CAF+IgG1.

Additional file 7. Figure 7: A) Western blot analysis of atrogin-1, MuRF-1 and CXCR2 expression in the TA muscle. B) Densitometry of atrogin-1, MuRF-1 and CXCR2 expression normalized by the expression of GAPDH. 4 mice per group were used for the experiments. The values were indicated as the mean ± SEM. *=p<0.05 and **=p<0.01 indicate significantly increased compared to IgG1 control. #=p<0.05 and ##=p<0.01 indicate significantly decreased compared to HCT 116 plus CAF+IgG1.

Additional file 8. Figure 8: A) Volcano plot showing gene expression changes in HCT-116+CAF injected mice compared with vehicle alone injected mice (designated as ‘Normal’), and HCT-116+CAF compared with HCT-116+CAF plus Neu Ab. B) Selected genes known to be implicated in skeletal muscle atrophy and/or ECM remodeling showing differential expression in HCT 116+CAF compared to Neu Ab. C-D) Western blot analysis and densitometry of AKT phosphorylation relative to AKT in C2C12 myotubes cultured in 20 ng/mL CXCL5 for 0.5 h, 1 h, and 2 h. For D) All experiments were performed 3 times independently and values are indicated as the mean ± SD. *=p<0.05 indicate significantly decreased compared to 0 h.

Additional file 9. Figure 9: A) ELISA detection of IL-6 in CCD-18Co human normal colon fibroblasts (NF) media, CAF CM, CAF CCM, and HCT 116 cancer cell CM. The CAF CM and CCM values are the mean of three sources of CAF: two derived from patients and one purchased commercially. B) Representative MYH2-stained images of C2C12 myotubes cultured with IL-6 for 72 h. (scale bar=150 μm). C) Calculation of mean myotube diameter. D) Representative MYH2-immunostained images of C2C12 myotubes cultured as follows: (1) differentiation media alone (DM alone) for 72 h; (2) 1 ng/mL of CXCL5 (CXCL5) for 72 h; (3) 2 ng/mL of IL-6 (IL-6) for 72 h; (4) Treatment of 1 ng/mL of CXCL5 plus 2 ng/mL of IL-6 (CXCL5+IL-6) for 72 h (scale bar=150 μm). E) Calculation of mean myotube diameter. F) Western blot analysis of MYH2 and atrogin-1 expression. G) Densitometry of MYH2 and atrogin-1 expression relative to GAPDH. H) Western blot analysis and densitometry of ERK1/2 and AKT phosphorylation in the C2C12 myotubes cultured with 1 ng/mL of CXCL5 plus 2 ng/mL of IL-6 for 0 h, 0.5 h, and 2 h. I) Densitometry of ERK1/2 and AKT phosphorylation. All experiments were performed 3 times independently and values were indicated as the mean ± SD. For A) **=p<0.01 indicate significantly increased compared to CAF CM. For C): **=p<0.01 and ***= p<0.001 indicate significantly decreased compared to DM alone. For E) and G): *=p<0.05 and **=p<0.01 indicate significantly decreased compared to DM alone. For I): **=p<0.01 and ***= p<0.001 indicate significantly decreased compared to 0 h incubation.

Additional file 10. Figure 10: qPCR analysis of atrogin-1, MuRF-1 and MyoG expression in the TA muscle of C57BL6/J mice treated with 40 ng/kg CXCL5 and 80 ng/kg IL-6 for 4 weeks, with or without 120 μg/kg CXCL5 neutralizing antibody. 120 μg/kg IgG1 was used as control. Gene expression levels were normalized by GAPDH expression. 4 mice per group were used for the experiments. The values are indicated as the mean ± SEM. *=p<0.05 and **=p<0.01 indicate significantly increased or decreased compared to IgG1 control. #=p<0.05 and ##=p<0.01 indicate significantly decreased compared to CXCL5+IL-6+IgG1

Additional file11. Figure 11: Immunohistochemical analysis of CXCL5 and vimentin (fibroblast marker) expression in tumor-stromal and normal tissues obtained from colon carcinoma patients. White arrows indicate overlapping CXCL5 and vimentin immunostaining.
