# Supplementary figures and images for: CXCL5 neutralization mitigates cancer cachexia by disrupting CAF-cancer cell crosstalk
Source: J Biomed Sci. 2025 Dec 15;32:107. doi: 10.1186/s12929-025-01192-0 (PMC12703897; doi:10.1186/s12929-025-01192-0)

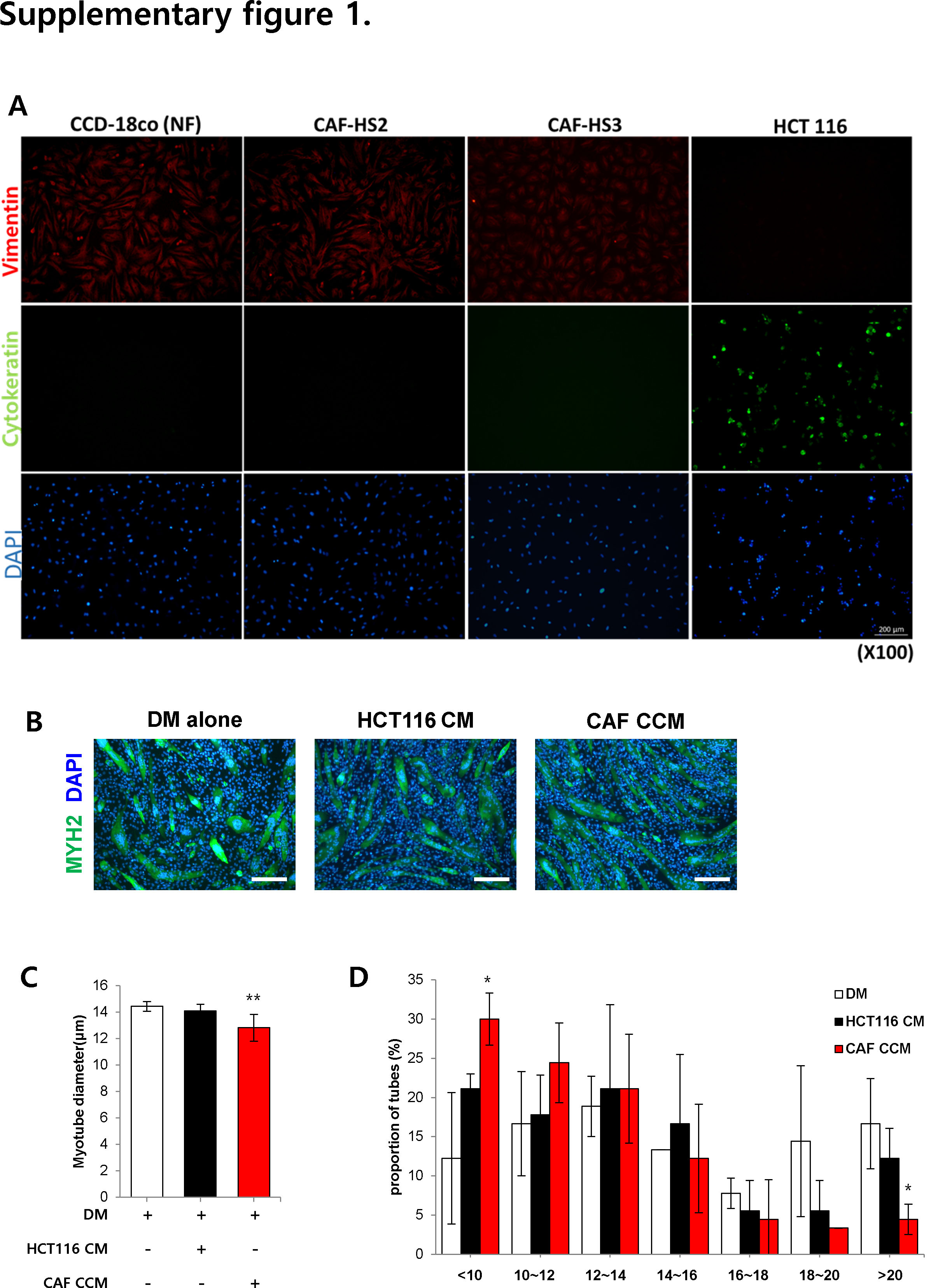

Supplement: Supplementary file 1 — Additional file 1: Figure 1. A) Immunostaining of vimentin (a fibroblast marker), and cytokeratin (an epithelial marker) in CCD-18Co normal colon fibroblasts (NF), colon carcinoma CAF purified from 2 patients (CAF-HS2 and CAF HS3), and HCT 116 cancer cells. B) Myosin heavy chain 2 (MYH2) immunostaining of C2C12 myotubes cultured in normal myotube differentiation media (DM) and treated with DM, HCT116 or CAF CCM for 72 h (scale bar=150 μm). C) Calculation of mean myotube diameter. D) Myotube diameter distribution (μm). *=p<0.05, **=p<0.01. [file 12929_2025_1192_MOESM1_ESM.tif]

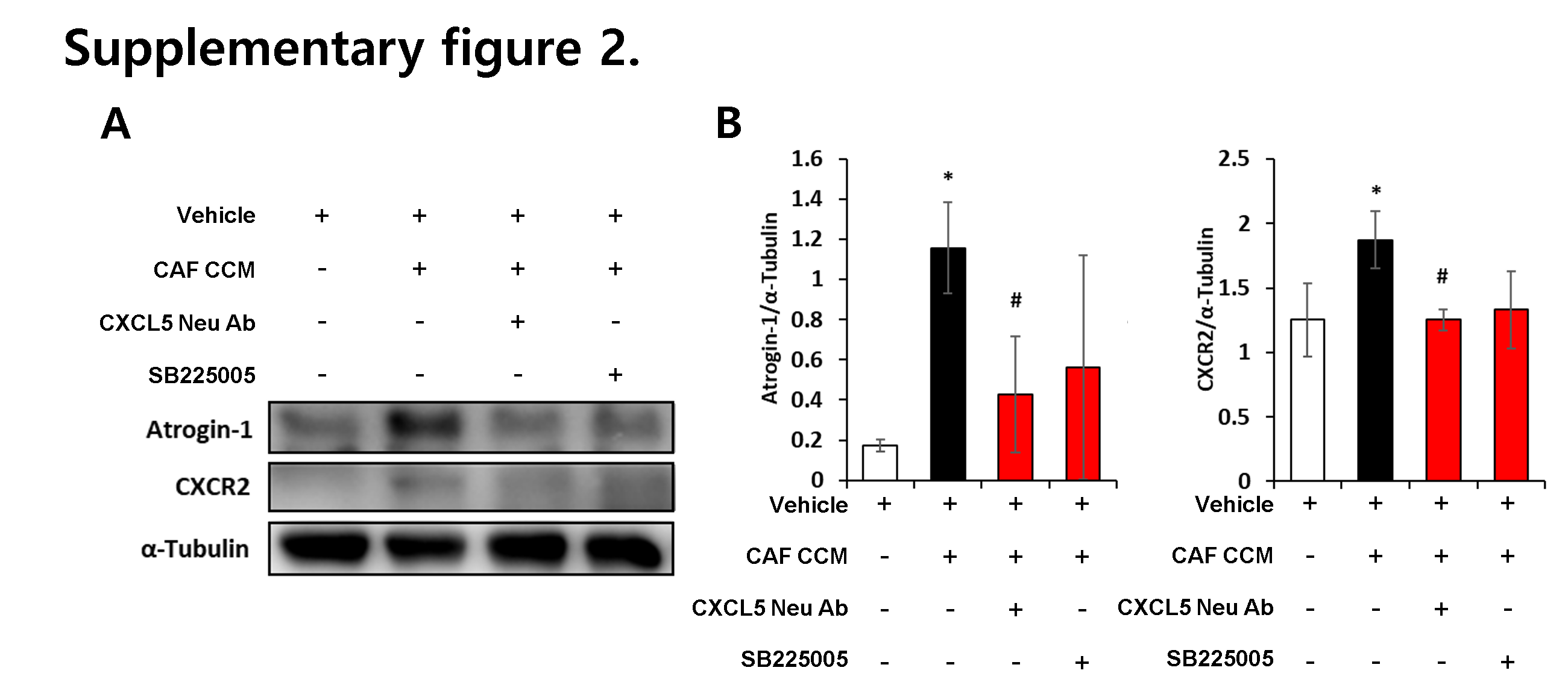

Supplement: Supplementary file 2 — Additional file 2: Figure 2: A) Western blot analysis of atrogin-1 and CXCR2 expression in C2C12 myotubes cultured in normal differentiation media with 0.1% DMSO (vehicle), CAF CCM and CAF CCM plus CXCL5 neutralizing antibody (0.5 μg/mL), or SB225005 (22 nM), an inhibitor of CXCR2, for 72h. α-Tubulin was used as a loading control. B) Densitometry of atrogin-1 and CXCR2 expression relative to α-tubulin. All experiments were performed 3 times independently and the values were indicated as the mean ± SD. *=p<0.05 indicate significantly increased compared to vehicle treated. #=p<0.05 indicate significantly decreased compared to CAF CCM treated. [file 12929_2025_1192_MOESM2_ESM.tif]

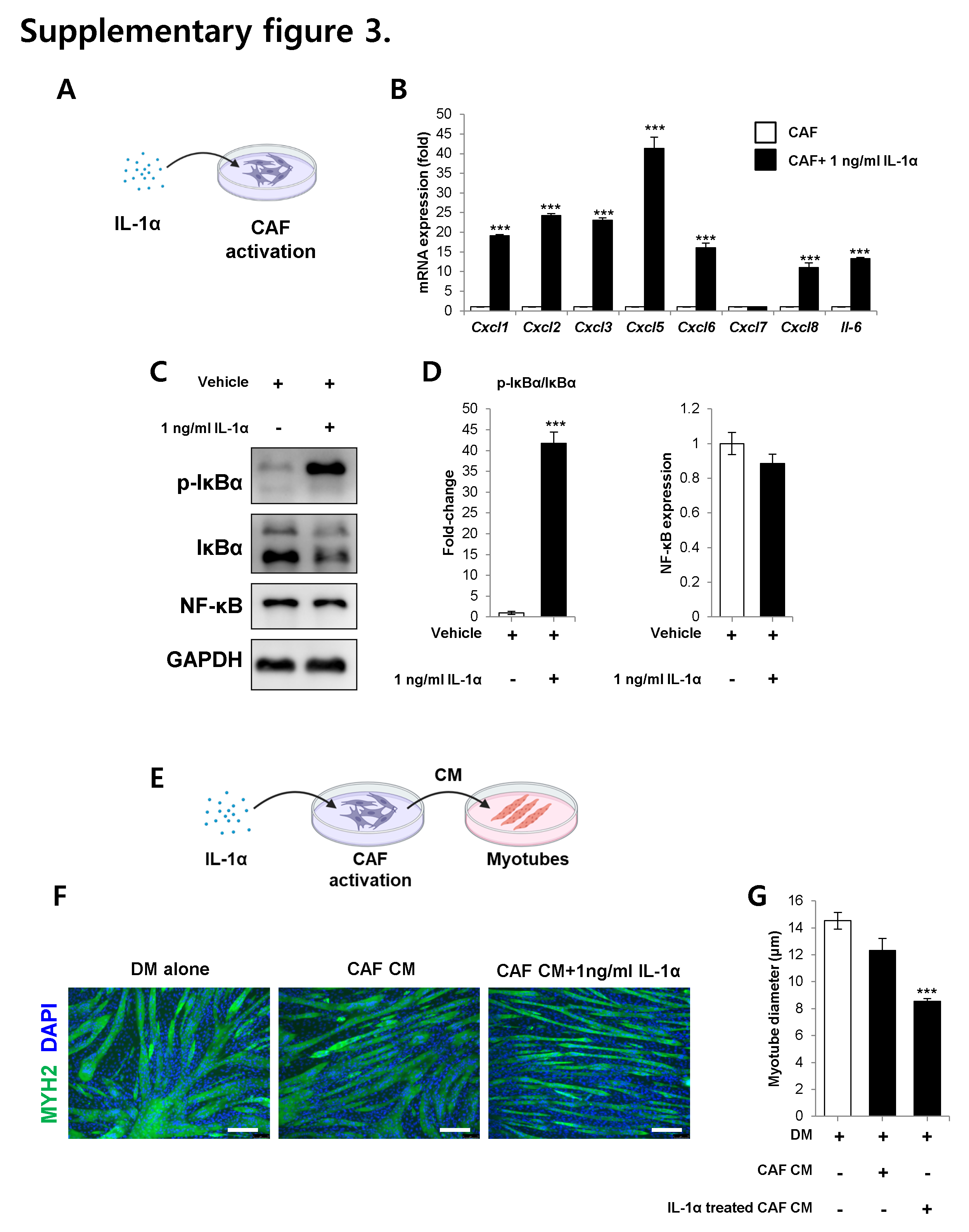

Supplement: Supplementary file 3 — Additional file 3. Figure 3: A) Schematic diagram of CAF activation by IL-1α treatment. B) qPCR analysis of CXCL1, 2, 3, 5, 6, 7, and 8, and IL-6 expression in IL-1α (1 ng/mL) treated CAF compared to CAF alone in serum free media with vehicle (0.1% BSA in PBS). C) Western blot analysis of IκBα phosphorylation and NF-κB expression. D) Densitometry of IκBα phosphorylation and NF-κB expression relative to IκB and GAPDH, respectively. E) Schematic diagram of induction of myotube atrophy by IL-1α-treated CAF. CAF CM was collected after IL-1α treatment (1 ng/mL) and incubated with C2C12 myotubes for 72 h. F) Representative MYH2-stained images of C2C12 myotubes cultured as follows: (1) Differentiation media (DM); (2) Treatment with non-stimulated CAF CM; (3) Treatment with IL-1α-stimulated CAF CM (scale bar = 100 μm). G) Calculation of mean myotube diameter. All experiments were performed 3 times independently and the values were indicated as the mean ± SD. ***= p<0.001 indicates significantly decreased compared to the non-stimulated CAF or DM alone group. [file 12929_2025_1192_MOESM3_ESM.tif]

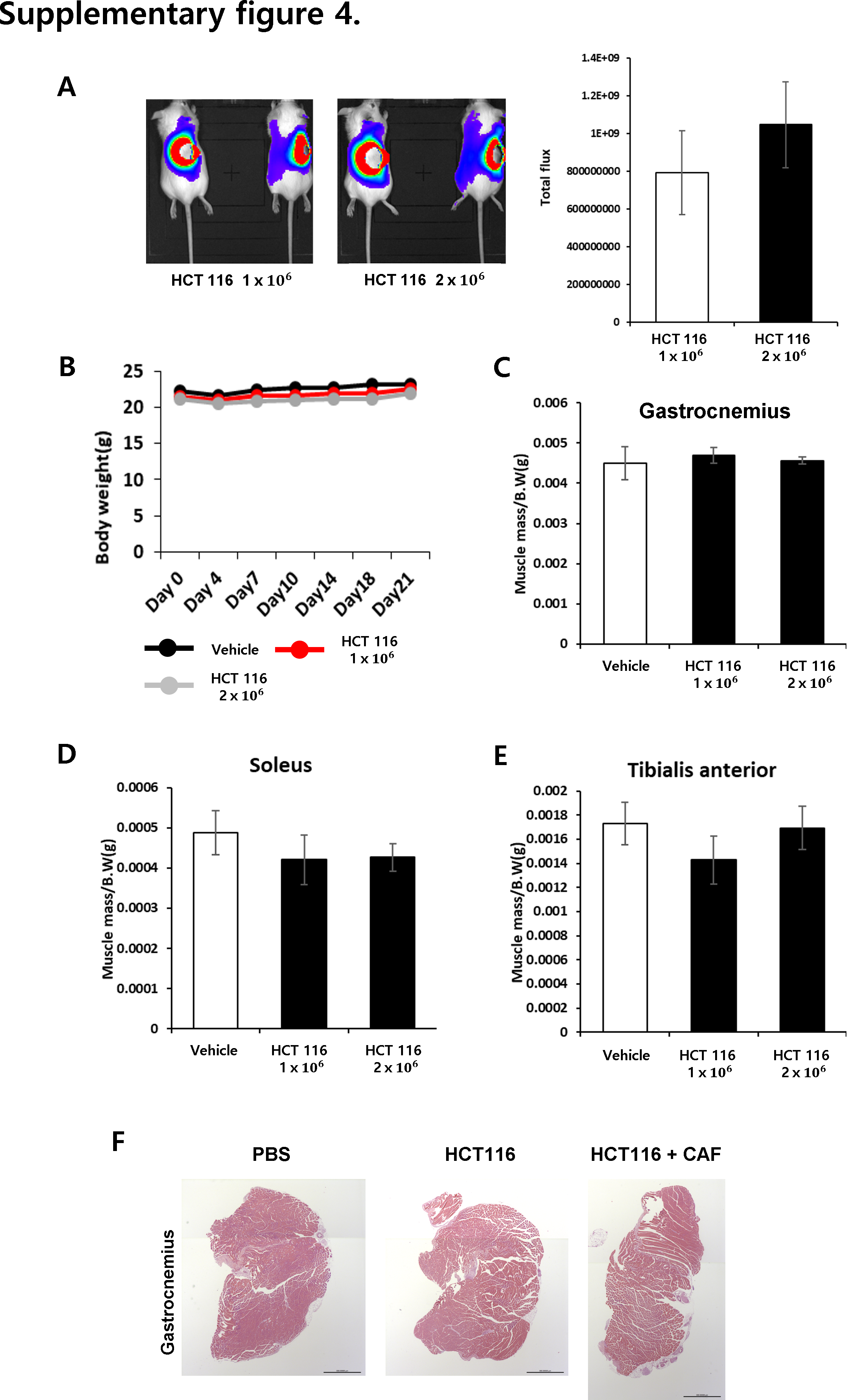

Supplement: Supplementary file 4 — Additional file 4. Figure 4: A) IVIS imaging of NOD-SCID mice 3 weeks post-xenograft with 1x10^6^ or 2x10^6^ HCT 116 cancer cells. Mean total flux detected from the tumor at the end point is also shown. B) Body weight changes for 3 weeks post-xenograft with 1x10^6^ or 2x10^6^ HCT 116 cancer cells. C-E) Gastrocnemius, soleus and TA muscle mass in vehicle alone-treated NOD-SCID (designated as ‘vehicle’) and NOD-SCID mice 3 weeks post-xenograft with 1x10^6^ or 2x10^6^ HCT 116 cancer cells. F) Low magnification image of the whole gastrocnemius muscle of NOD-SCID mice 3 weeks post-xenograft with 1x10^6^ human HCT 116 luc2 colon cancer cells, or 1x10^6^ HCT 116 luc2 cancer cells plus 2x10^6^ human colon CAF (scale bar=1 mm). 3 mice per group were used for the experiments and the values were indicated as the mean ± SEM. [file 12929_2025_1192_MOESM4_ESM.tif]

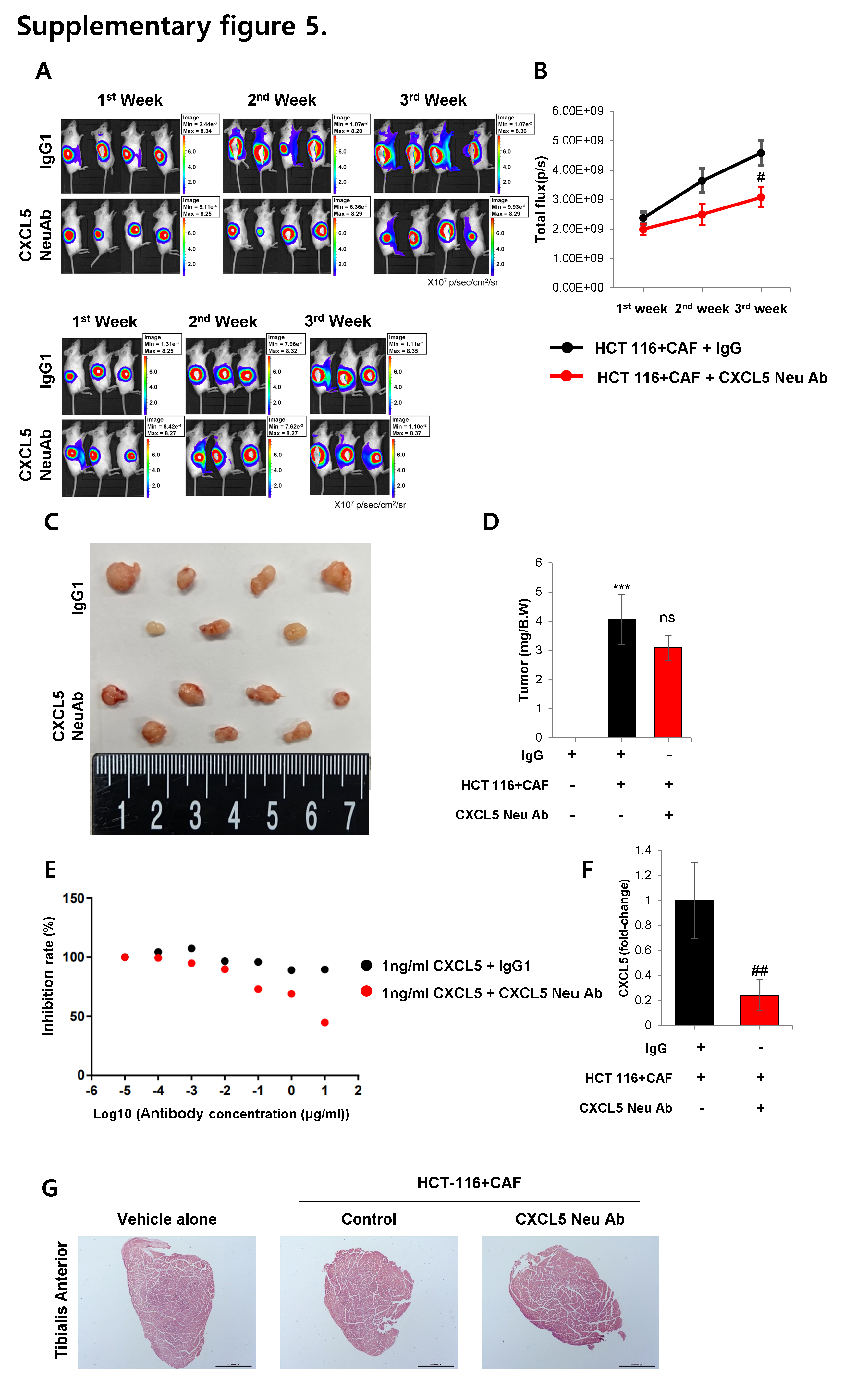

Supplement: Supplementary file 5 — Additional file 5. Figure 5: A) IVIS imaging of NOD-SCID mice at 3 weeks post-xenograft with human HCT 116 luc2 cancer cells plus human colon CAF, with or without CXCL5 neutralization (CXCL5 Neu Ab) and 120 μg/kg IgG1 used as control. B) Mean total flux detected from the tumor at the 3 week end point. C-D) Dissected tumors and tumor mass. E) CXCL5 neutralization was tested with CXCL5 ELISA results on cell free condition. F) CXCL5 concentration in the collected serum. G) Low magnification image of the whole tibialis anterior muscle of NOD-SCID mice 3 weeks post-xenograft with human HCT 116 luc2 cancer cells plus human colon CAF, with or without CXCL5 neutralization (CXCL5 Neu Ab) (scale bar=1 mm). For A-D and F), 7 mice per group were used for the experiments and the values were indicated as the mean ± SEM. *=p<0.05 and **=p<0.01 indicate significantly increased or decreased compared to IgG1 control. #=p<0.05 and ##=p<0.01 indicate significantly decreased compared to HCT116 plus CAF+IgG1. [file 12929_2025_1192_MOESM5_ESM.tif]

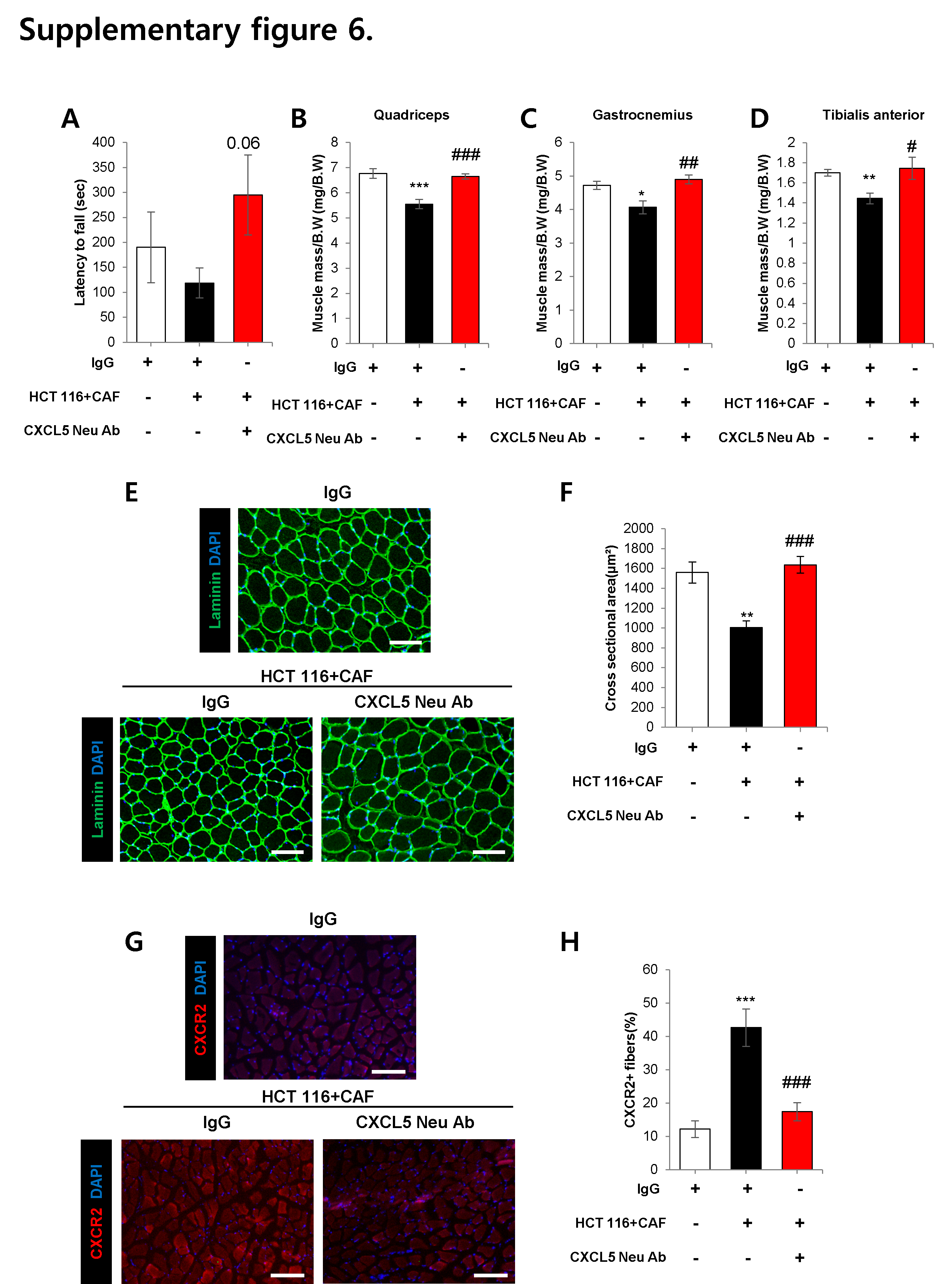

Supplement: Supplementary file 6 — Additional file 6. Figure 6: A) Hanging tolerance in the treated mice. B) Quadriceps muscle mass. C) Gastrocnemius muscle mass. D) Tibialis anterior (TA) muscle mass. E) Laminin staining of the TA muscle (scale bar=150 µm). F) TA myofiber cross sectional area. G) CXCR2 staining of the TA muscle (scale bar=150 µm). H) The proportion of CXCR2 positive fibers in TA muscle. 7 mice per group were used for the experiments and the values are indicated as the mean ± SEM. *=p<0.05 and **=p<0.01 indicate significantly decreased compared to IgG1 control. #=p<0.05 and ##=p<0.01 indicate significantly increased compared to HCT 116 plus CAF+IgG1. [file 12929_2025_1192_MOESM6_ESM.tif]

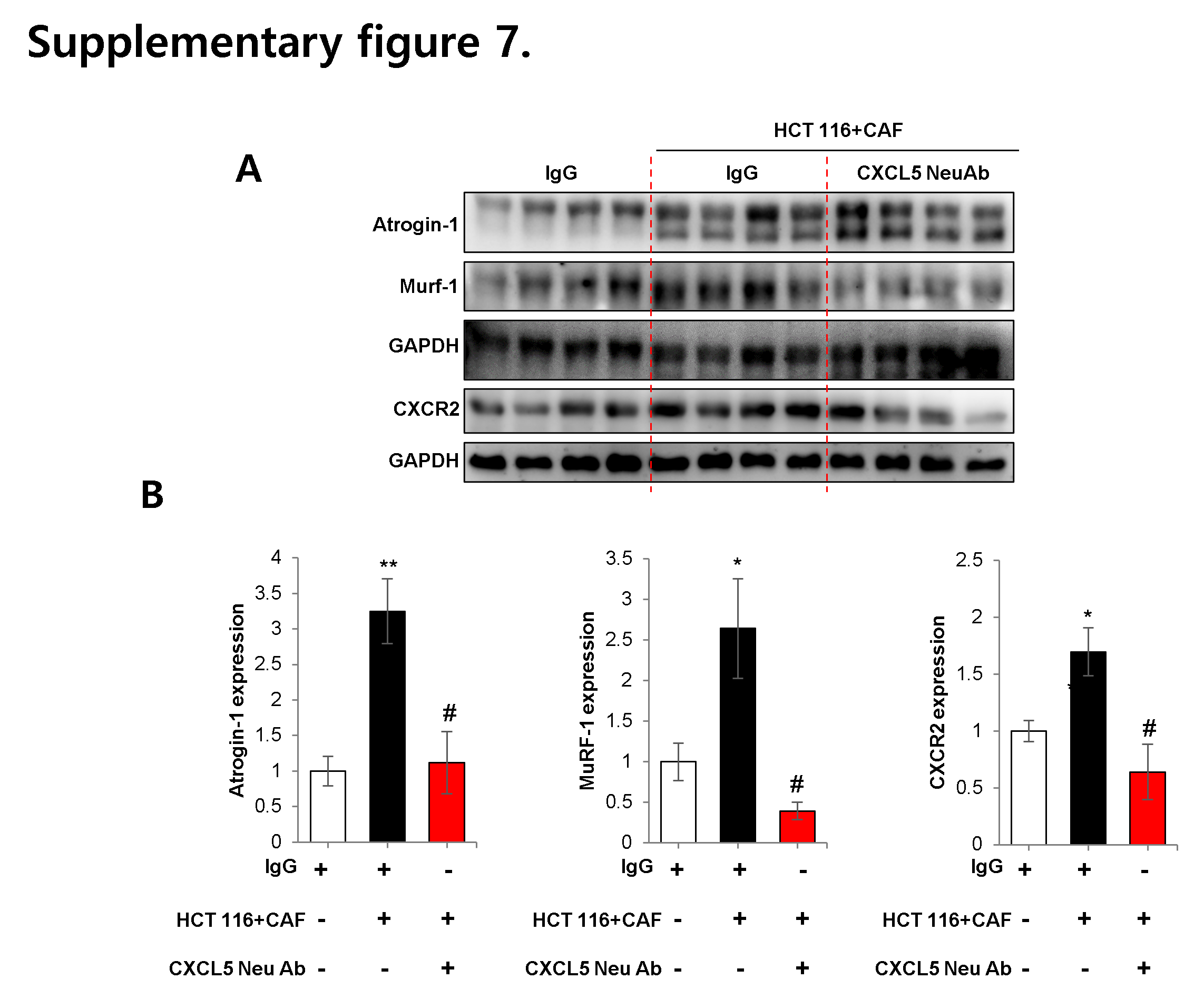

Supplement: Supplementary file 7 — Additional file 7. Figure 7: A) Western blot analysis of atrogin-1, MuRF-1 and CXCR2 expression in the TA muscle. B) Densitometry of atrogin-1, MuRF-1 and CXCR2 expression normalized by the expression of GAPDH. 4 mice per group were used for the experiments. The values were indicated as the mean ± SEM. *=p<0.05 and **=p<0.01 indicate significantly increased compared to IgG1 control. #=p<0.05 and ##=p<0.01 indicate significantly decreased compared to HCT 116 plus CAF+IgG1. [file 12929_2025_1192_MOESM7_ESM.tif]

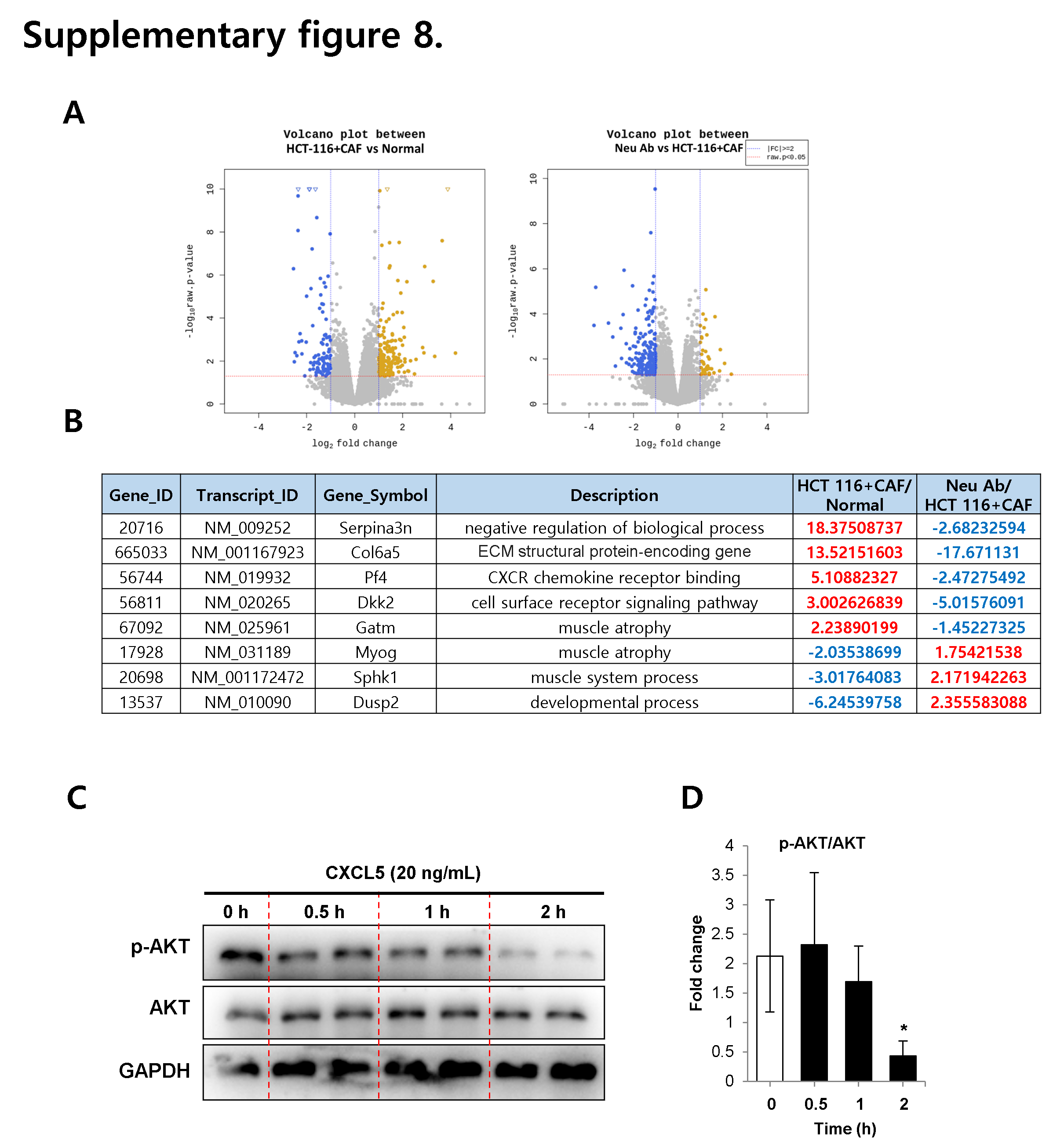

Supplement: Supplementary file 8 — Additional file 8. Figure 8: A) Volcano plot showing gene expression changes in HCT-116+CAF injected mice compared with vehicle alone injected mice (designated as ‘Normal’), and HCT-116+CAF compared with HCT-116+CAF plus Neu Ab. B) Selected genes known to be implicated in skeletal muscle atrophy and/or ECM remodeling showing differential expression in HCT 116+CAF compared to Neu Ab. C-D) Western blot analysis and densitometry of AKT phosphorylation relative to AKT in C2C12 myotubes cultured in 20 ng/mL CXCL5 for 0.5 h, 1 h, and 2 h. For D) All experiments were performed 3 times independently and values are indicated as the mean ± SD. *=p<0.05 indicate significantly decreased compared to 0 h. [file 12929_2025_1192_MOESM8_ESM.tif]

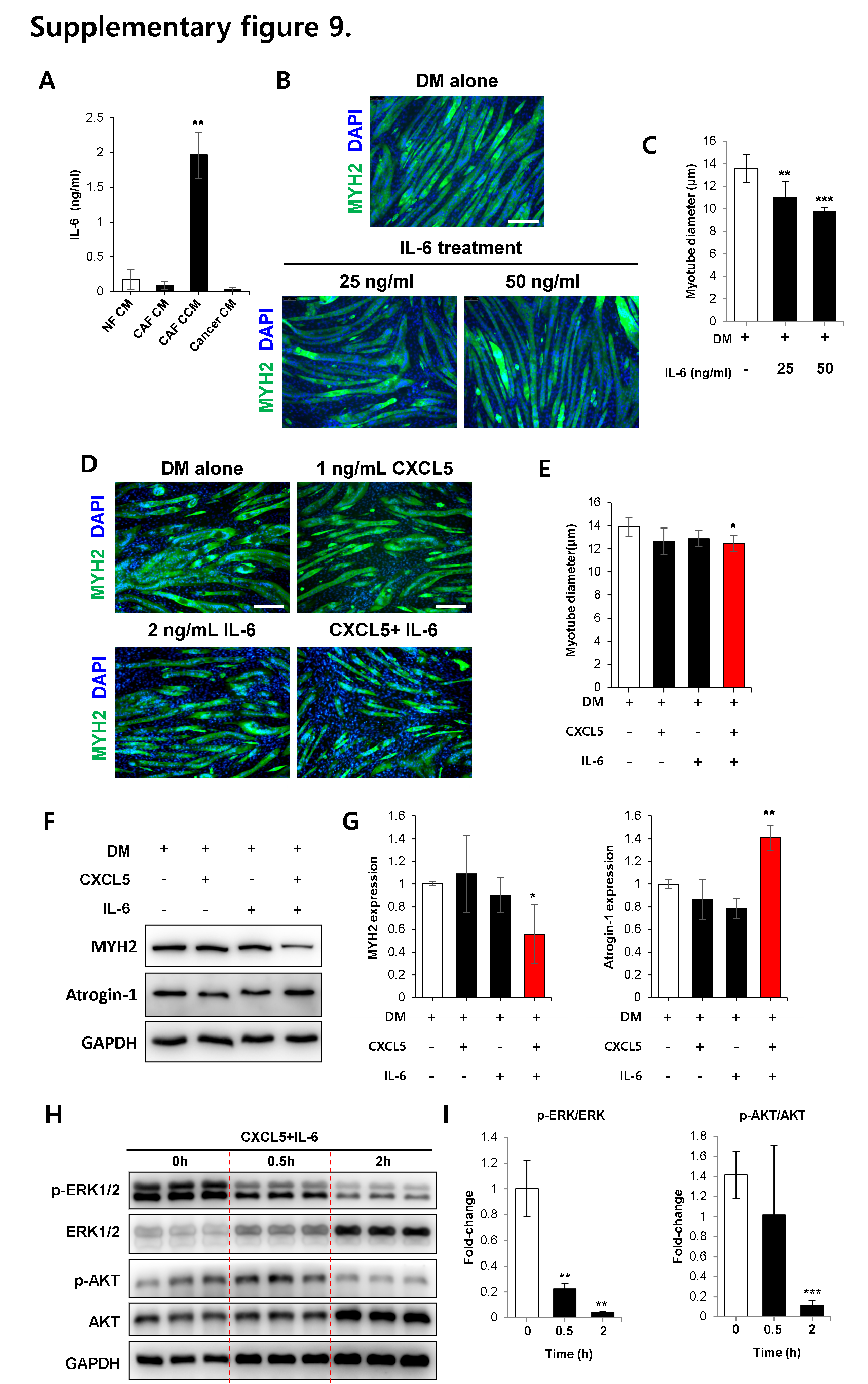

Supplement: Supplementary file 9 — Additional file 9. Figure 9: A) ELISA detection of IL-6 in CCD-18Co human normal colon fibroblasts (NF) media, CAF CM, CAF CCM, and HCT 116 cancer cell CM. The CAF CM and CCM values are the mean of three sources of CAF: two derived from patients and one purchased commercially. B) Representative MYH2-stained images of C2C12 myotubes cultured with IL-6 for 72 h. (scale bar=150 μm). C) Calculation of mean myotube diameter. D) Representative MYH2-immunostained images of C2C12 myotubes cultured as follows: (1) differentiation media alone (DM alone) for 72 h; (2) 1 ng/mL of CXCL5 (CXCL5) for 72 h; (3) 2 ng/mL of IL-6 (IL-6) for 72 h; (4) Treatment of 1 ng/mL of CXCL5 plus 2 ng/mL of IL-6 (CXCL5+IL-6) for 72 h (scale bar=150 μm). E) Calculation of mean myotube diameter. F) Western blot analysis of MYH2 and atrogin-1 expression. G) Densitometry of MYH2 and atrogin-1 expression relative to GAPDH. H) Western blot analysis and densitometry of ERK1/2 and AKT phosphorylation in the C2C12 myotubes cultured with 1 ng/mL of CXCL5 plus 2 ng/mL of IL-6 for 0 h, 0.5 h, and 2 h. I) Densitometry of ERK1/2 and AKT phosphorylation. All experiments were performed 3 times independently and values were indicated as the mean ± SD. For A) **=p<0.01 indicate significantly increased compared to CAF CM. For C): **=p<0.01 and ***= p<0.001 indicate significantly decreased compared to DM alone. For E) and G): *=p<0.05 and **=p<0.01 indicate significantly decreased compared to DM alone. For I): **=p<0.01 and ***= p<0.001 indicate significantly decreased compared to 0 h incubation. [file 12929_2025_1192_MOESM9_ESM.tif]

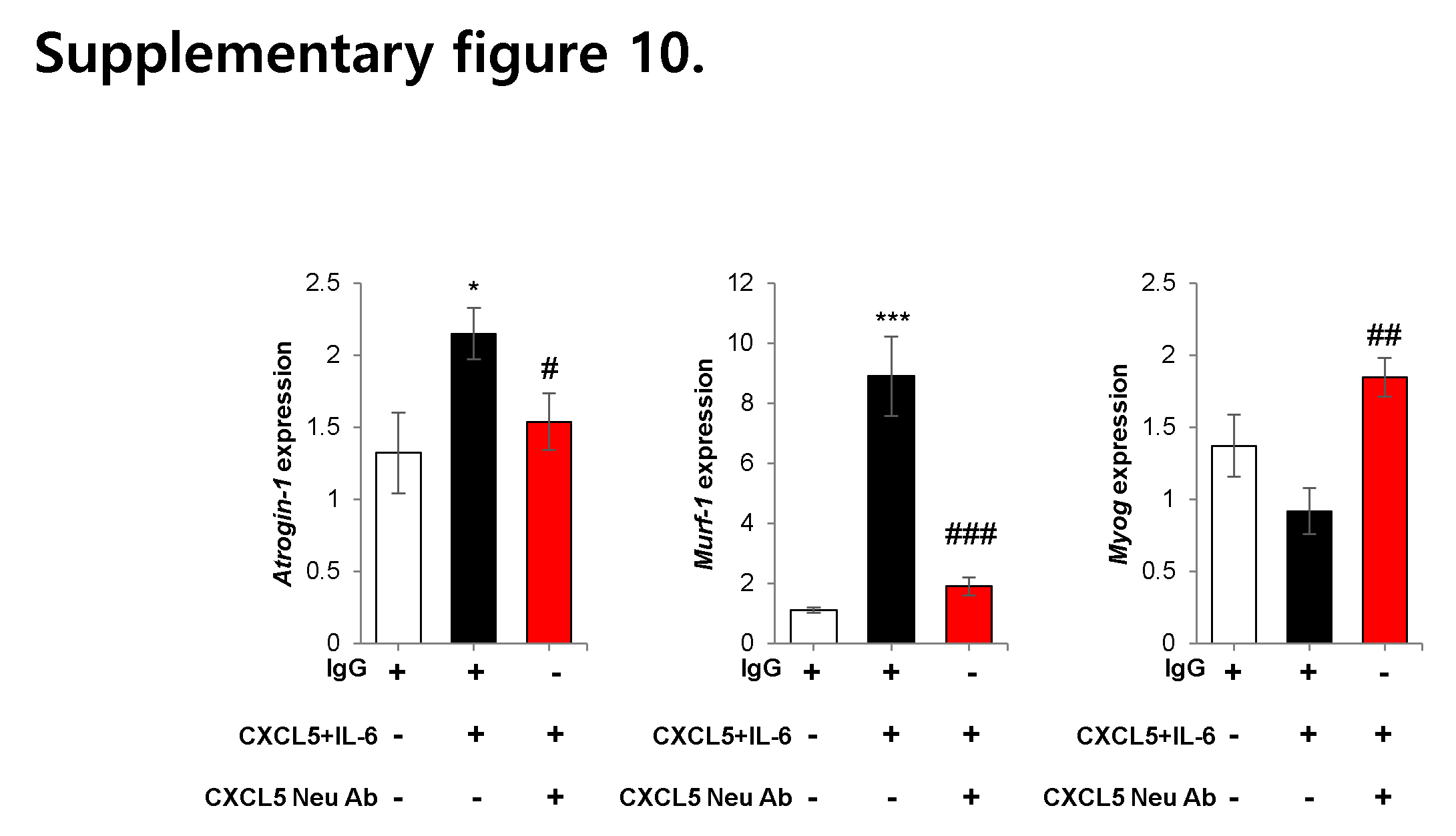

Supplement: Supplementary file 10 — Additional file 10. Figure 10: qPCR analysis of atrogin-1, MuRF-1 and MyoG expression in the TA muscle of C57BL6/J mice treated with 40 ng/kg CXCL5 and 80 ng/kg IL-6 for 4 weeks, with or without 120 μg/kg CXCL5 neutralizing antibody. 120 μg/kg IgG1 was used as control. Gene expression levels were normalized by GAPDH expression. 4 mice per group were used for the experiments. The values are indicated as the mean ± SEM. *=p<0.05 and **=p<0.01 indicate significantly increased or decreased compared to IgG1 control. #=p<0.05 and ##=p<0.01 indicate significantly decreased compared to CXCL5+IL-6+IgG1 [file 12929_2025_1192_MOESM10_ESM.tif]

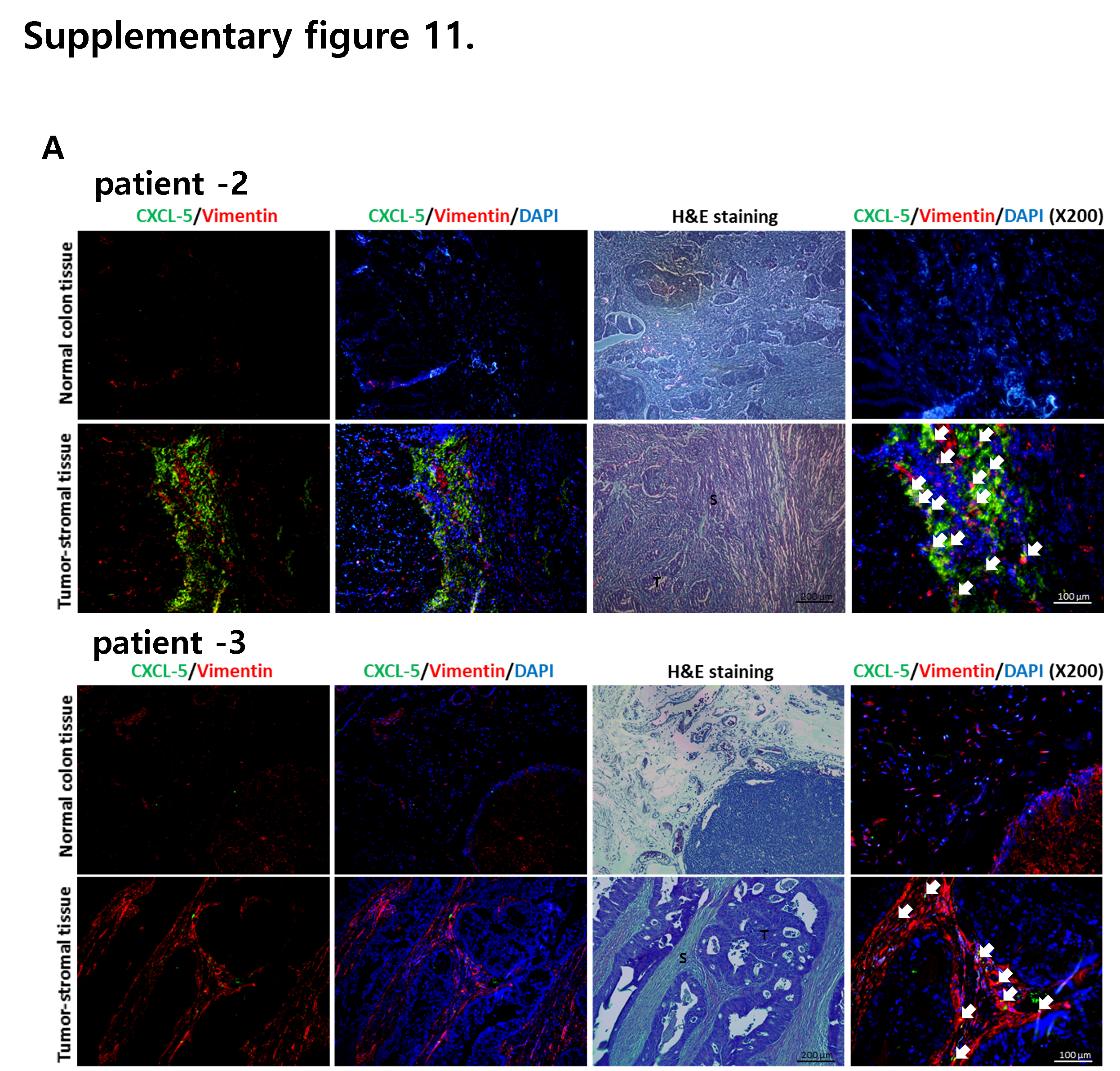

Supplement: Supplementary file 11 — Additional file11. Figure 11: Immunohistochemical analysis of CXCL5 and vimentin (fibroblast marker) expression in tumor-stromal and normal tissues obtained from colon carcinoma patients. White arrows indicate overlapping CXCL5 and vimentin immunostaining. [file 12929_2025_1192_MOESM11_ESM.tif]
